# Supplementary material for: L-Type Ca2+ Channel Inhibition Rescues the LPS-Induced Neuroinflammatory Response and Impairments in Spatial Memory and Dendritic Spine Formation
Source: Int J Mol Sci. 2022 Nov 6;23(21):13606. doi: 10.3390/ijms232113606 (PMC9655622; doi:10.3390/ijms232113606)
Supplement: Supplementary file 1 [file ijms-23-13606-s001.zip › ijms-1927373-supplementary.pdf]

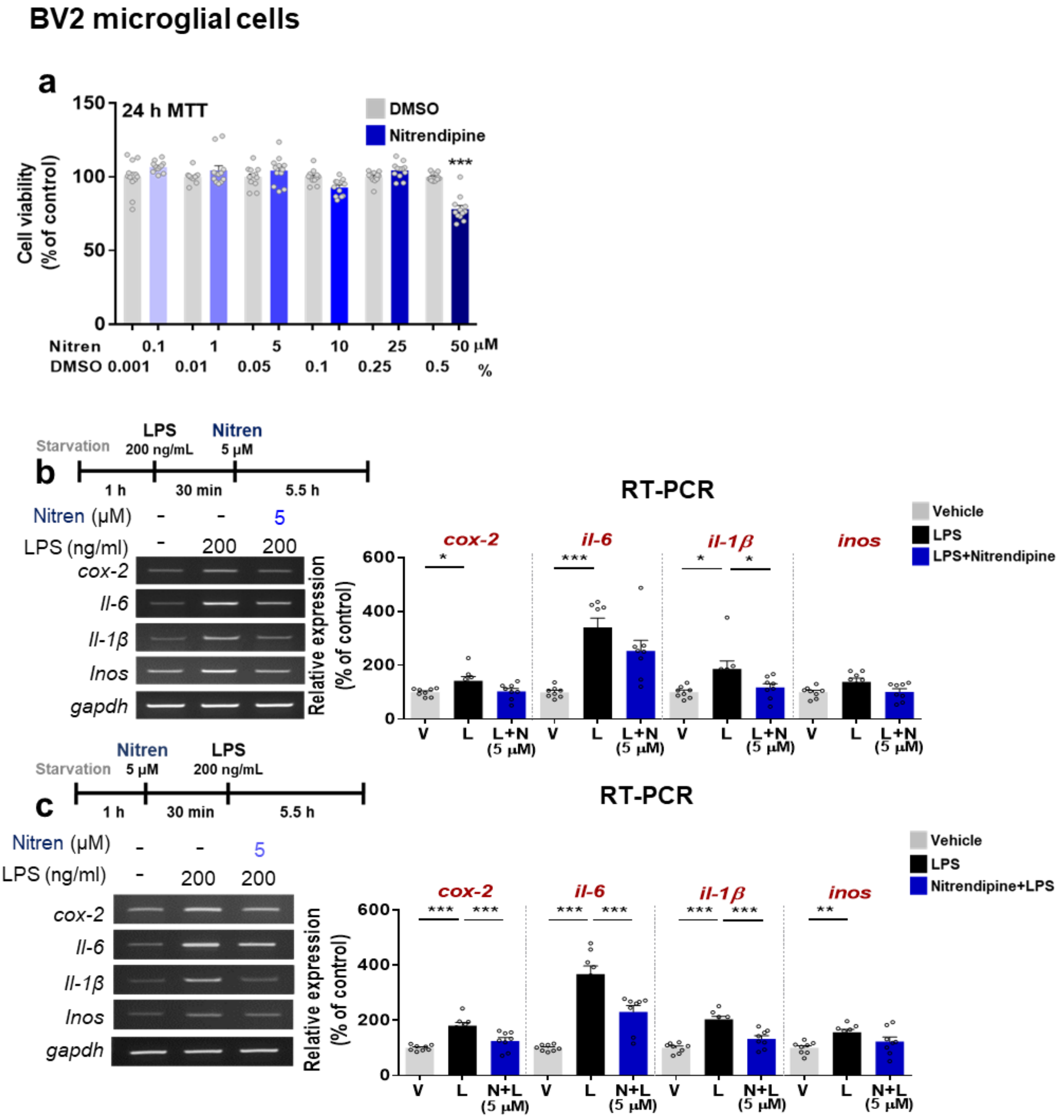

Supplementary Figure S1. The L-type Ca^{2+} channel blocker nitrendipine decreases LPS-induced proinflammatory cytokine levels in BV2 microglial cells. **(a)** Cell viability was measured by the MTT assay (n = 12/group). **(b)** Cells were treated with LPS (200 ng/ml) or PBS followed by nitrendipine (5 μM) or vehicle (1% DMSO) as shown, and RT-PCR was conducted (n = 8/group). **(c)** Cells were treated with nitrendipine (5 μM) or vehicle (1% DMSO) followed by LPS (200 ng/ml) or PBS as shown, and RT-PCR was conducted (n = 8/group). $*p < 0.05$, $**p < 0.01$, $***p < 0.001$, V: Vehicle; L: LPS; N or Nitren: Nitrendipine.

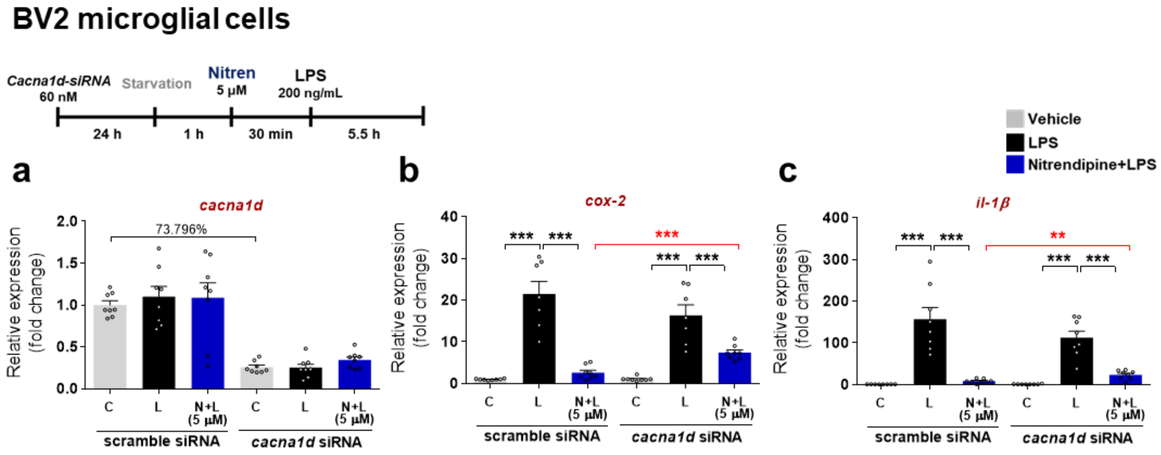

Supplementary Figure S2. The reduction of LPS-induced proinflammatory cytokine *cox-2* and *il-1β* mRNA levels by 5 μM nitrendipine is partially dependent on the L-type Ca^{2+} channel. (**a**) Real-time PCR analysis of *cacna1d* gene expression in cells treated with nitrendipine (5 μM) or vehicle (1% DMSO) followed by LPS (200 ng/ml) or PBS as shown (n = 8/group). (**b-c**) Real-time PCR analysis of *cox-2* and *il-1β* mRNA levels in cells transfected with *cacna1d* siRNA (60 nM) or scramble (control) siRNA for 24 h and subsequently treated with nitrendipine (5 μM) or vehicle (1% DMSO) followed by LPS (200 ng/ml) or PBS as shown (n = 8/group). $**p < 0.01$, $***p < 0.001$, V: Vehicle; L: LPS; N or Nitren: Nitrendipine.

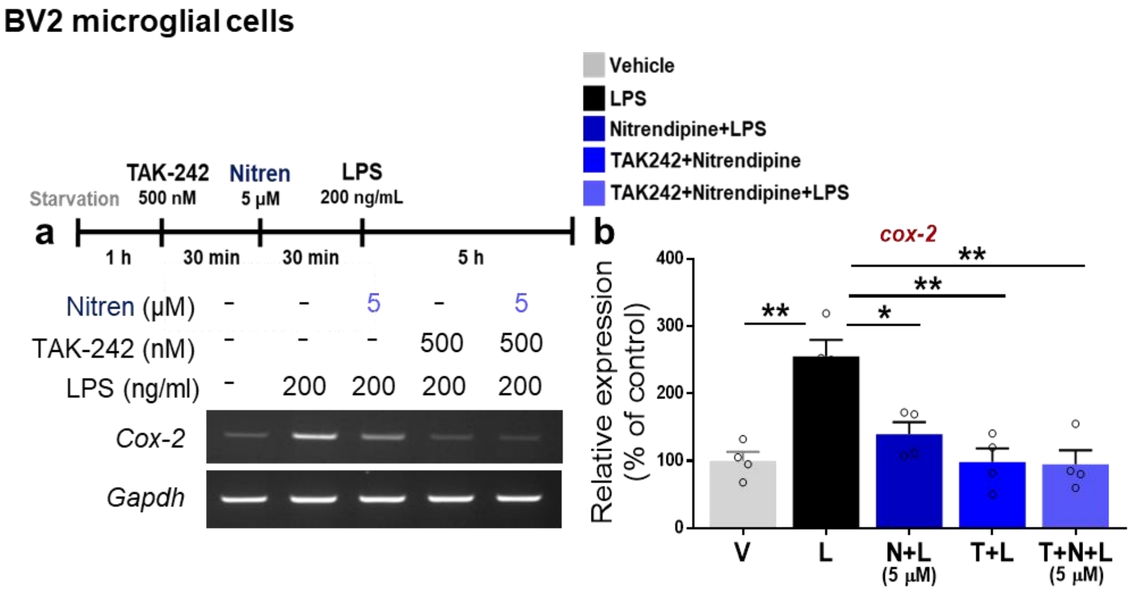

Supplementary Figure S3. Nitrendipine downregulates LPS-stimulated proinflammatory responses through TLR4 signaling in BV2 microglial cells. (**a**) RT-PCR analysis of proinflammatory cytokine *cox-2* expression in cells treated as shown with a TLR4 inhibitor (TAK-242, 500 nM), nitrendipine (5 μM), and LPS (200 ng/ml). (**b**) Quantification of data from **a** (n= 4/group). **p* < 0.05, ***p* < 0.01, V: Vehicle; L: LPS; N or Nitren: Nitrendipine.

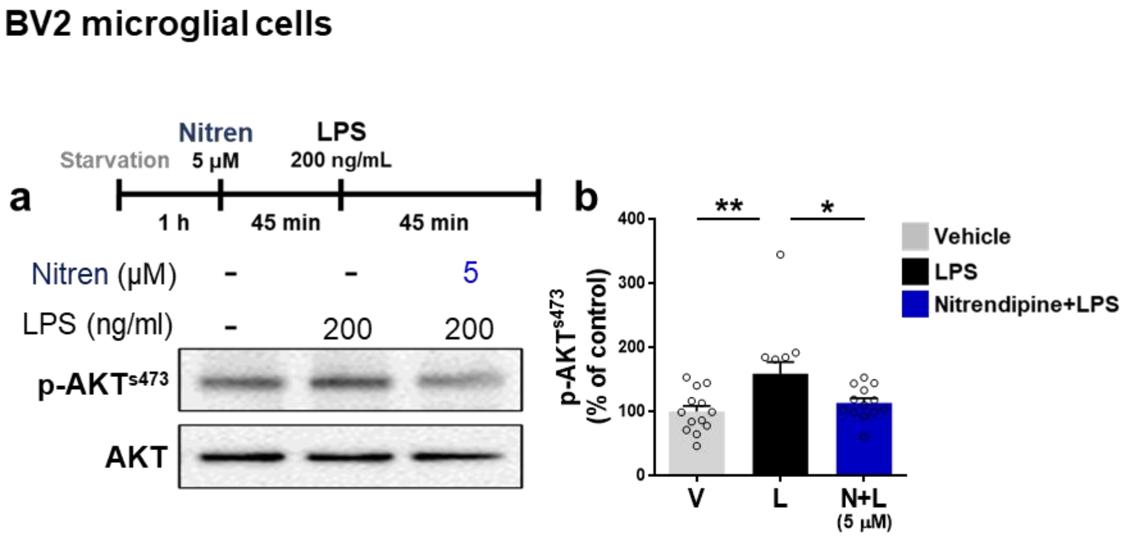

Supplementary Figure S4. Nitrendipine reduces LPS-mediated AKT phosphorylation in BV2 microglial cells. **(a)** Western blot analysis of AKT phosphorylation in cells treated with nitrendipine (5 μM) or vehicle (1% DMSO) followed by LPS (200 ng/ml) or PBS as shown. (b) Quantification of data from **a** (n = 13/group). *p < 0.05, **p < 0.01, V: Vehicle; L: LPS; N or Nitren: Nitrendipine.

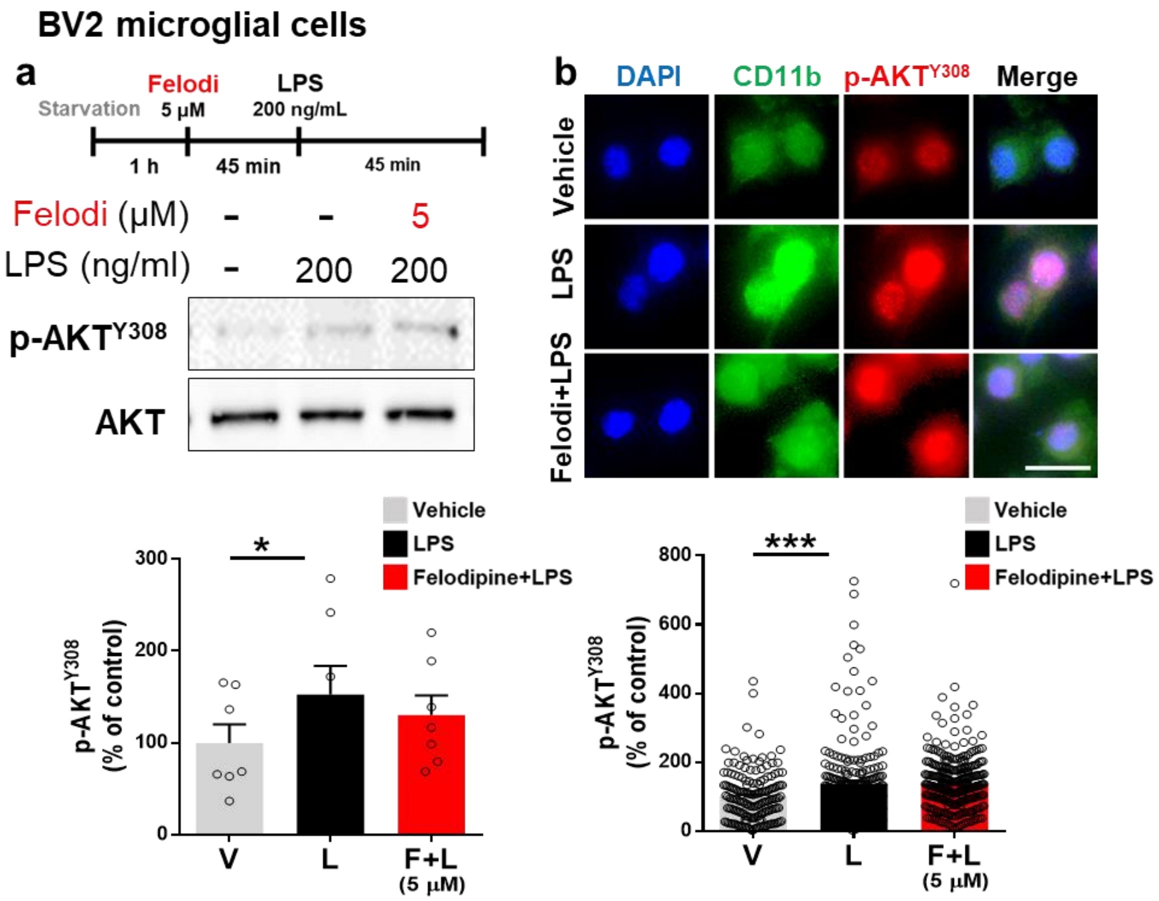

Supplementary Figure S5. Felodipine does not alter LPS-induced p-AKT^{Y308} levels in BV2 microglial cells. (**a)** Western blotting analysis of p-AKT^{Y308} and AKT levels in cells treated with felodipine (5 μM) or vehicle (1% DMSO) followed by LPS (200 ng/ml) or PBS as shown (n = 7/group). (**b)** Immunocytochemistry analysis of p-AKT^{Y308} expression in cells treated with felodipine (5 μM) or vehicle (1% DMSO) followed by LPS (200 ng/ml) or PBS (Veh, n = 171; LPS, n = 229; Felodi +LPS, n = 321). *$p < 0.05$, ***$p < 0.001$ Scale bar: 20 μm, V: Vehicle; L: LPS; F or Felodi: Felodipine.

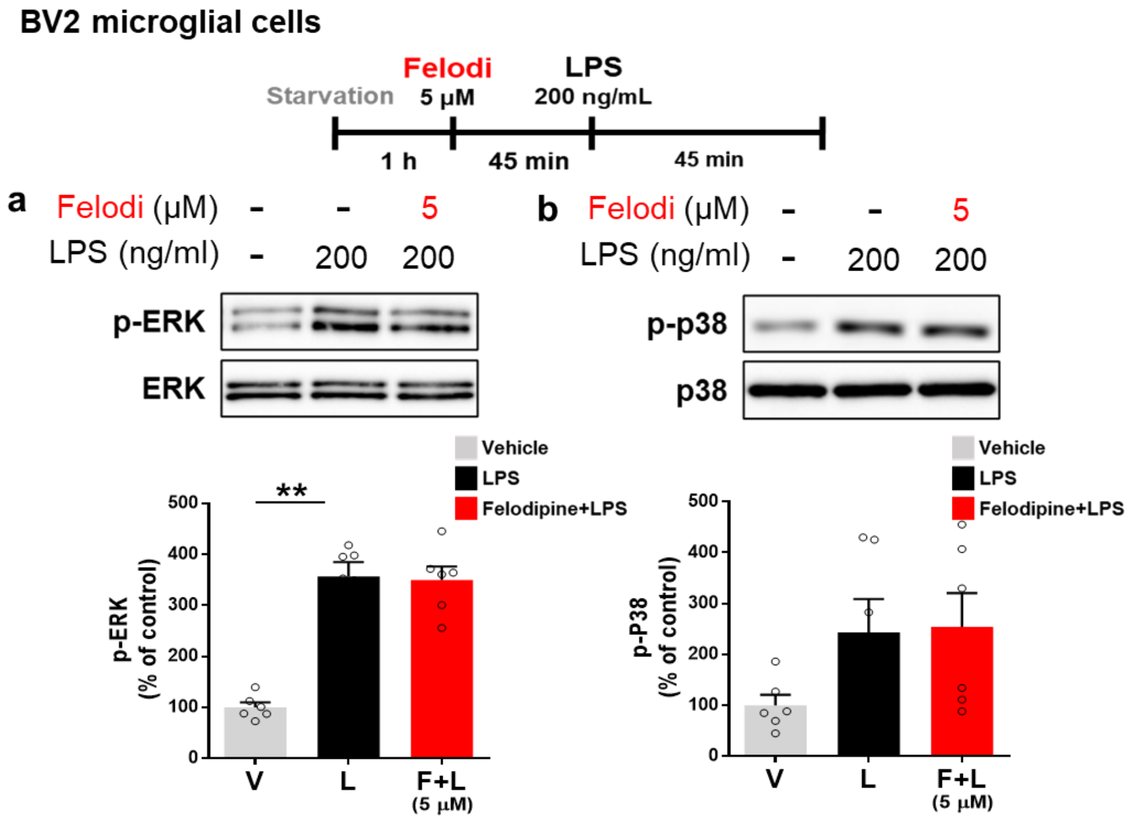

Supplementary Figure S6. Felodipine does not alter LPS-evoked ERK/P38 signaling in BV2 microglial cells. (**a, b)** Western blot analysis of p-ERK and ERK levels (**a**) or p-P38 and P38 levels (**b**) in cells treated with felodipine (5 µM) or vehicle (1% DMSO) followed by LPS (200 ng/ml) or PBS as shown (n = 6/group). **$p < 0.01$ V: Vehicle; L: LPS; F or Felodi: Felodipine.

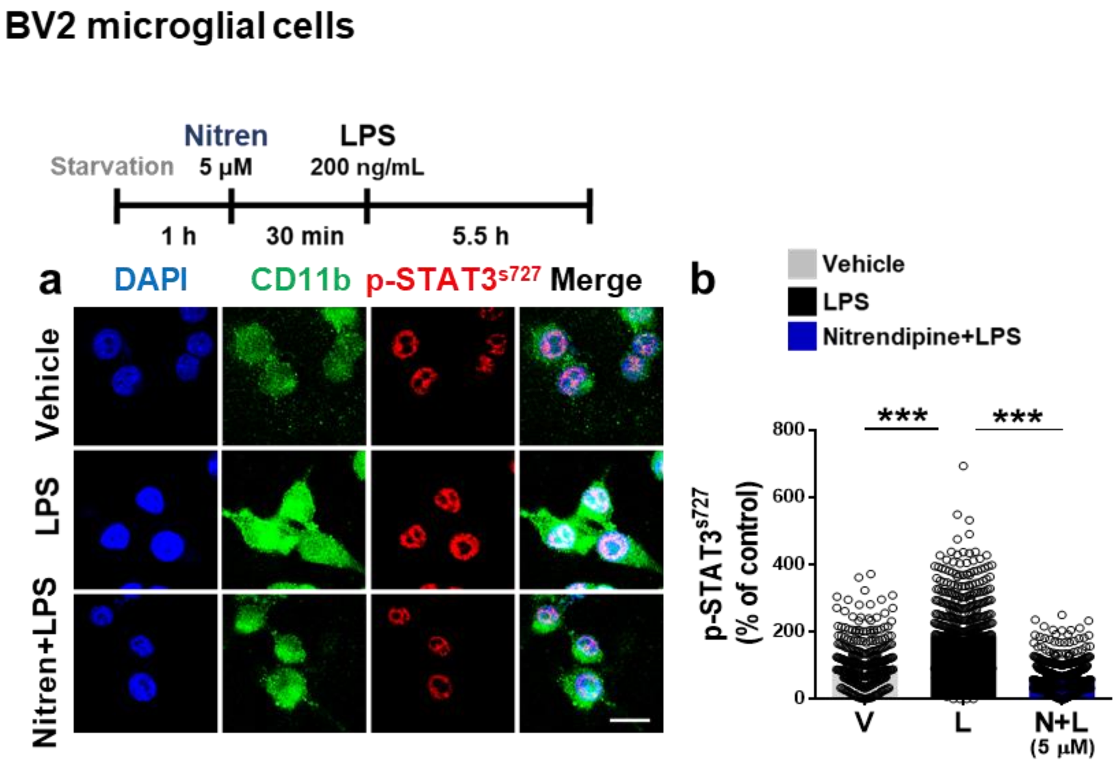

Supplementary Figure S7. Nitrendipine decreases LPS-induced STAT3 phosphorylation in BV2 microglial cells. **(a)** Immunocytochemical staining of p-STAT3 in cells treated with nitrendipine (5 μM) or vehicle (1% DMSO) followed by LPS (200 ng/ml) or PBS as shown. (**b**) Quantification of data from **a** (Veh n = 392; LPS n = 581; Nitren+LPS n = 522). ***$p < 0.001$, Scale bar: 20 μm, V: Vehicle; L: LPS; N or Nitren: Nitrendipine.

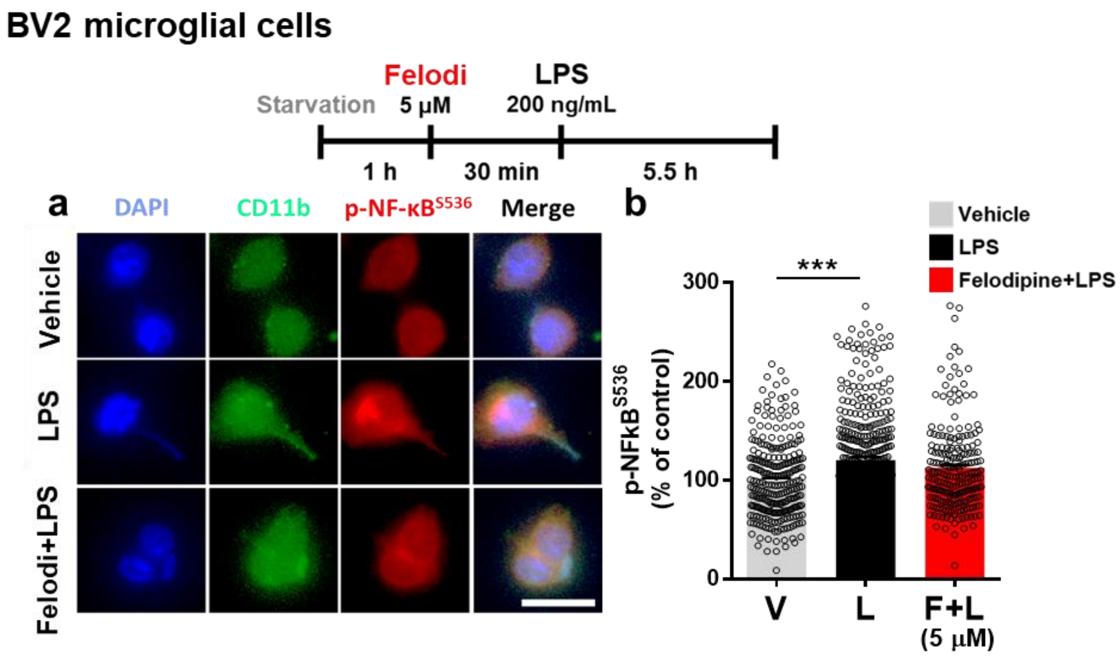

Supplementary Figure S8. Felodipine pre-treatment does not affect LPS-evoked nuclear NF-kB phosphorylation in BV2 microglial cells. **(a)** Results of immunocytochemistry analysis of p-NF-kB and CD11b expression in cells treated with felodipine (5 μM) or vehicle (1% DMSO) and with LPS (200 ng/ml) or PBS as shown. **(b)** Quantification of data from **a** (Veh n = 267; LPS n = 361; Felodi +LPS n = 213). Scale bar = 20 μm, ***$p < 0.001$, V: Vehicle; L: LPS; F or Felodi: Felodipine.

Supplementary Table S1. One-way ANOVA (Tukey's test) and significance of the results of the *in vitro* experiments in this study.

Figure 1b 24 h MTT						
Number of families	1					
Number of comparisons per family	66					
Alpha	0.05					
Tukey's multiple comparisons test	Mean Diff.	95.00% CI of diff.	Significant?	Summary	Adjusted P Value	
DMSO (0.001%) vs. S1885 0.1 uM	2.319	-1.053 to 5.691	No	ns	0.4708	A-B
DMSO (0.001%) vs. DMSO (0.01%)	5.714e-006	-3.372 to 3.372	No	ns	>0.9999	A-C
DMSO (0.001%) vs. S1885 1 uM	-0.04332	-3.415 to 3.329	No	ns	>0.9999	A-D
DMSO (0.001%) vs. DMSO (0.05%)	1.429e-006	-3.372 to 3.372	No	ns	>0.9999	A-E
DMSO (0.001%) vs. S1885 5 uM	0.8458	-2.526 to 4.218	No	ns	0.9994	A-F
DMSO (0.001%) vs. DMSO (0.1%)	1.143e-005	-3.372 to 3.372	No	ns	>0.9999	A-G
DMSO (0.001%) vs. S1885 10 uM	-0.4741	-3.846 to 2.898	No	ns	>0.9999	A-H
DMSO (0.001%) vs. DMSO (0.25%)	-1.429e-006	-3.372 to 3.372	No	ns	>0.9999	A-I
DMSO (0.001%) vs. S1885 25 uM	5.223	1.851 to 8.595	Yes	****	<0.0001	A-J
DMSO (0.001%) vs. DMSO (0.5%)	1.143e-005	-3.372 to 3.372	No	ns	>0.9999	A-K
DMSO (0.001%) vs. S1885 50 uM	85.38	82.01 to 88.75	Yes	****	<0.0001	A-L
S1885 0.1 uM vs. DMSO (0.01%)	-2.319	-5.691 to 1.053	No	ns	0.4708	B-C
S1885 0.1 uM vs. S1885 1 uM	-2.362	-5.734 to 1.01	No	ns	0.4418	B-D
S1885 0.1 uM vs. DMSO (0.05%)	-2.319	-5.691 to 1.053	No	ns	0.4708	B-E
S1885 0.1 uM vs. S1885 5 uM	-1.473	-4.845 to 1.899	No	ns	0.9425	B-F
S1885 0.1 uM vs. DMSO (0.1%)	-2.319	-5.691 to 1.053	No	ns	0.4708	B-G
S1885 0.1 uM vs. S1885 10 uM	-2.793	-6.165 to 0.5793	No	ns	0.2033	B-H
S1885 0.1 uM vs. DMSO (0.25%)	-2.319	-5.691 to 1.053	No	ns	0.4708	B-I
S1885 0.1 uM vs. S1885 25 uM	2.904	-0.4676 to 6.276	No	ns	0.1598	B-J
S1885 0.1 uM vs. DMSO (0.5%)	-2.319	-5.691 to 1.053	No	ns	0.4708	B-K
S1885 0.1 uM vs. S1885 50 uM	83.06	79.69 to 86.44	Yes	****	<0.0001	B-L
DMSO (0.01%) vs. S1885 1 uM	-0.04332	-3.415 to 3.329	No	ns	>0.9999	C-D
DMSO (0.01%) vs. DMSO (0.05%)	-4.286e-006	-3.372 to 3.372	No	ns	>0.9999	C-E
DMSO (0.01%) vs. S1885 5 uM	0.8458	-2.526 to 4.218	No	ns	0.9994	C-F
DMSO (0.01%) vs. DMSO (0.1%)	5.714e-006	-3.372 to 3.372	No	ns	>0.9999	C-G
DMSO (0.01%) vs. S1885 10 uM	-0.4741	-3.846 to 2.898	No	ns	>0.9999	C-H
DMSO (0.01%) vs. DMSO (0.25%)	-7.143e-006	-3.372 to 3.372	No	ns	>0.9999	C-I
DMSO (0.01%) vs. S1885 25 uM	5.223	1.851 to 8.595	Yes	****	<0.0001	C-J
DMSO (0.01%) vs. DMSO (0.5%)	5.714e-006	-3.372 to 3.372	No	ns	>0.9999	C-K
DMSO (0.01%) vs. S1885 50 uM	85.38	82.01 to 88.75	Yes	****	<0.0001	C-L
S1885 1 uM vs. DMSO (0.05%)	0.04332	-3.329 to 3.415	No	ns	>0.9999	D-E
S1885 1 uM vs. S1885 5 uM	0.8891	-2.483 to 4.261	No	ns	0.9990	D-F
S1885 1 uM vs. DMSO (0.1%)	0.04333	-3.329 to 3.415	No	ns	>0.9999	D-G
S1885 1 uM vs. S1885 10 uM	-0.4308	-3.803 to 2.941	No	ns	>0.9999	D-H
S1885 1 uM vs. DMSO (0.25%)	0.04332	-3.329 to 3.415	No	ns	>0.9999	D-I
S1885 1 uM vs. S1885 25 uM	5.266	1.894 to 8.638	Yes	****	<0.0001	D-J
S1885 1 uM vs. DMSO (0.5%)	0.04333	-3.329 to 3.415	No	ns	>0.9999	D-K
S1885 1 uM vs. S1885 50 uM	85.43	82.05 to 88.8	Yes	****	<0.0001	D-L
DMSO (0.05%) vs. S1885 5 uM	0.8458	-2.526 to 4.218	No	ns	0.9994	E-F
DMSO (0.05%) vs. DMSO (0.1%)	1e-005	-3.372 to 3.372	No	ns	>0.9999	E-G
DMSO (0.05%) vs. S1885 10 uM	-0.4741	-3.846 to 2.898	No	ns	>0.9999	E-H
DMSO (0.05%) vs. DMSO (0.25%)	-2.857e-006	-3.372 to 3.372	No	ns	>0.9999	E-I
DMSO (0.05%) vs. S1885 25 uM	5.223	1.851 to 8.595	Yes	****	<0.0001	E-J
DMSO (0.05%) vs. DMSO (0.5%)	1e-005	-3.372 to 3.372	No	ns	>0.9999	E-K
DMSO (0.05%) vs. S1885 50 uM	85.38	82.01 to 88.75	Yes	****	<0.0001	E-L
S1885 5 uM vs. DMSO (0.1%)	-0.8458	-4.218 to 2.526	No	ns	0.9994	F-G
S1885 5 uM vs. S1885 10 uM	-1.32	-4.692 to 2.052	No	ns	0.9734	F-H
S1885 5 uM vs. DMSO (0.25%)	-0.8458	-4.218 to 2.526	No	ns	0.9994	F-I
S1885 5 uM vs. S1885 25 uM	4.377	1.005 to 7.749	Yes	**	0.0022	F-J
S1885 5 uM vs. DMSO (0.5%)	-0.8458	-4.218 to 2.526	No	ns	0.9994	F-K
S1885 5 uM vs. S1885 50 uM	84.54	81.16 to 87.91	Yes	****	<0.0001	F-L
DMSO (0.1%) vs. S1885 10 uM	-0.4741	-3.846 to 2.898	No	ns	>0.9999	G-H
DMSO (0.1%) vs. DMSO (0.25%)	-1.286e-005	-3.372 to 3.372	No	ns	>0.9999	G-I
DMSO (0.1%) vs. S1885 25 uM	5.223	1.851 to 8.595	Yes	****	<0.0001	G-J
DMSO (0.1%) vs. DMSO (0.5%)	0	-3.372 to 3.372	No	ns	>0.9999	G-K
DMSO (0.1%) vs. S1885 50 uM	85.38	82.01 to 88.75	Yes	****	<0.0001	G-L
S1885 10 uM vs. DMSO (0.25%)	0.4741	-2.898 to 3.846	No	ns	>0.9999	H-I
S1885 10 uM vs. S1885 25 uM	5.697	2.325 to 9.069	Yes	****	<0.0001	H-J

S1885 10 uM vs. DMSO (0.5%)	0.4741	-2.898 to 3.846	No	ns	>0.9999	H-K
S1885 10 uM vs. S1885 50 uM	85.86	82.48 to 89.23	Yes	****	<0.0001	H-L
DMSO (0.25%) vs. S1885 25 uM	5.223	1.851 to 8.595	Yes	****	<0.0001	I-J
DMSO (0.25%) vs. DMSO (0.5%)	1.286e-005	-3.372 to 3.372	No	ns	>0.9999	I-K
DMSO (0.25%) vs. S1885 50 uM	85.38	82.01 to 88.75	Yes	****	<0.0001	I-L
S1885 25 uM vs. DMSO (0.5%)	-5.223	-8.595 to -1.851	Yes	****	<0.0001	J-K
S1885 25 uM vs. S1885 50 uM	80.16	76.79 to 83.53	Yes	****	<0.0001	J-L
DMSO (0.5%) vs. S1885 50 uM	85.38	82.01 to 88.75	Yes	****	<0.0001	K-L

Figure 1c Real-time PCR: cox-2

Number of families	1					
Number of comparisons per family	10					
Alpha	0.05					
Tukey's multiple comparisons test	Mean Diff.	95.00% CI of diff.	Significant?	Summary	Adjusted P Value	
V vs. L	-41.88	-52.87 to -30.88	Yes	****	<0.0001	A-B
V vs. L+F 1μM	-18.61	-29.99 to -7.223	Yes	***	0.0004	A-C
V vs. L+F 2.5μM	-8.233	-19.23 to 2.764	No	ns	0.2210	A-D
V vs. L+F 5μM	-1.639	-12.64 to 9.358	No	ns	0.9926	A-E
L vs. L+F 1μM	23.27	11.89 to 34.65	Yes	****	<0.0001	B-C
L vs. L+F 2.5μM	33.64	22.65 to 44.64	Yes	****	<0.0001	B-D
L vs. L+F 5μM	40.24	29.24 to 51.23	Yes	****	<0.0001	B-E
L+F 1μM vs. L+F 2.5μM	10.37	-1.01 to 21.76	No	ns	0.0882	C-D
L+F 1μM vs. L+F 5μM	16.97	5.583 to 28.35	Yes	**	0.0012	C-E
L+F 2.5μM vs. L+F 5μM	6.594	-4.403 to 17.59	No	ns	0.4319	D-E

Figure 1d RT-PCR: cox-2

Number of families	1							
Number of comparisons per family	3							
Alpha	0.05							
Tukey's multiple comparisons test	Mean Diff.	95.00% CI of diff.	Significant?	Summary	Adjusted P Value			
V vs. L	-68.2	-108.9 to -27.53	Yes	**	0.0011	A-B		
V vs. L+F	-25.34	-66 to 15.33	No	ns	0.2800	A-C		
L vs. L+F	42.86	2.192 to 83.52	Yes	*	0.0377	B-C		
Test details	Mean 1	Mean 2	Mean Diff.	SE of diff.	n1	n2	q	DF
V vs. L	100	168.2	-68.2	16.13	8	8	5.978	21
V vs. L+F	100	125.3	-25.34	16.13	8	8	2.221	21
L vs. L+F	168.2	125.3	42.86	16.13	8	8	3.757	21

Figure 1d RT-PCR: il-6

Number of families	1							
Number of comparisons per family	3							
Alpha	0.05							
Tukey's multiple comparisons test	Mean Diff.	95.00% CI of diff.	Significant?	Summary	Adjusted P Value			
V vs. L	-167.9	-221.9 to -114	Yes	****	<0.0001	E-F		
V vs. L+F	-121.9	-175.9 to -67.94	Yes	****	<0.0001	E-G		
L vs. L+F	46.05	-7.907 to 100	No	ns	0.1036	F-G		
Test details	Mean 1	Mean 2	Mean Diff.	SE of diff.	n1	n2	q	DF
V vs. L	100	267.9	-167.9	21.41	8	8	11.1	21
V vs. L+F	100	221.9	-121.9	21.41	8	8	8.053	21
L vs. L+F	267.9	221.9	46.05	21.41	8	8	3.042	21

Figure 1d RT-PCR: il-1β

Number of families	1							
Number of comparisons per family	3							
Alpha	0.05							
Tukey's multiple comparisons test	Mean Diff.	95.00% CI of diff.	Significant?	Summary	Adjusted P Value			
V vs. L	-55.01	-93.07 to -16.95	Yes	**	0.0042	I-J		
V vs. L+F	-43	-81.06 to -4.936	Yes	*	0.0251	I-K		
L vs. L+F	12.01	-26.05 to 50.07	No	ns	0.7098	J-K		
Test details	Mean 1	Mean 2	Mean Diff.	SE of diff.	n1	n2	q	DF
V vs. L	100	155	-55.01	15.1	8	8	5.152	21

V vs. L+F	100	143	-43	15.1	8	8	4.027	21
L vs. L+F	155	143	12.01	15.1	8	8	1.125	21

Figure 1d RT-PCR: inos

Number of families	1							
Number of comparisons per family	3							
Alpha	0.05							
Tukey's multiple comparisons test	Mean Diff.	95.00% CI of diff.	Significant?	Summary	Adjusted P Value			
V vs. L	-51.94	-82.12 to -21.75	Yes	***	0.0008	M-N		
V vs. L+F	-29.19	-59.38 to 0.9975	No	ns	0.0592	M-O		
L vs. L+F	22.75	-7.44 to 52.93	No	ns	0.1636	N-O		
Test details	Mean 1	Mean 2	Mean Diff.	SE of diff.	n1	n2	q	DF
V vs. L	100	151.9	-51.94	11.98	8	8	6.133	21
V vs. L+F	100	129.2	-29.19	11.98	8	8	3.447	21
L vs. L+F	151.9	129.2	22.75	11.98	8	8	2.686	21

Figure 1e ELISA: COX-2

Number of families	1							
Number of comparisons per family	3							
Alpha	0.05							
Tukey's multiple comparisons test	Mean Diff.	95.00% CI of diff.	Significant?	Summary	Adjusted P Value			
V vs. L	-1587	-2760 to -414	Yes	**	0.0086	A-B		
V vs. L+F	-20.96	-1194 to 1152	No	ns	0.9988	A-C		
L vs. L+F	1566	393 to 2739	Yes	**	0.0094	B-C		
Test details	Mean 1	Mean 2	Mean Diff.	SE of diff.	n1	n2	q	DF
V vs. L	777.3	2364	-1587	448.1	8	8	5.008	14
V vs. L+F	777.3	798.3	-20.96	448.1	8	8	0.06615	14
L vs. L+F	2364	798.3	1566	448.1	8	8	4.942	14

Figure 1f Real-time PCR: cox-2

Number of families	1					
Number of comparisons per family	10					
Alpha	0.05					
Tukey's multiple comparisons test	Mean Diff.	95.00% CI of diff.	Significant?	Summary	Adjusted P Value	
V vs. L	-31.26	-46.62 to -15.9	Yes	****	<0.0001	A-B
V vs. F 1μM+L	-13.75	-29.11 to 1.612	No	ns	0.0976	A-C
V vs. F 2.5μM+L	-5.558	-20.92 to 9.805	No	ns	0.8350	A-D
V vs. F 5μM+L	-0.2205	-15.58 to 15.14	No	ns	>0.9999	A-E
L vs. F 1μM+L	17.51	2.147 to 32.87	Yes	*	0.0188	B-C
L vs. F 2.5μM+L	25.7	10.34 to 41.07	Yes	***	0.0003	B-D
L vs. F 5μM+L	31.04	15.68 to 46.4	Yes	****	<0.0001	B-E
F 1μM+L vs. F 2.5μM+L	8.194	-7.17 to 23.56	No	ns	0.5485	C-D
F 1μM+L vs. F 5μM+L	13.53	-1.832 to 28.89	No	ns	0.1064	C-E
F 2.5μM+L vs. F 5μM+L	5.337	-10.03 to 20.7	No	ns	0.8541	D-E

Figure 1f Real-time PCR: il-1β

Number of families	1					
Number of comparisons per family	10					
Alpha	0.05					
Tukey's multiple comparisons test	Mean Diff.	95.00% CI of diff.	Significant?	Summary	Adjusted P Value	
V vs. L	-267.7	-351.5 to -183.8	Yes	****	<0.0001	A-B
V vs. F 1μM+L	-88.14	-172 to -4.301	Yes	*	0.0353	A-C
V vs. F 2.5μM+L	-25.08	-108.9 to 58.76	No	ns	0.9094	A-D
V vs. F 5μM+L	-3.708	-87.55 to 80.13	No	ns	>0.9999	A-E
L vs. F 1μM+L	179.5	95.7 to 263.4	Yes	****	<0.0001	B-C
L vs. F 2.5μM+L	242.6	158.8 to 326.4	Yes	****	<0.0001	B-D
L vs. F 5μM+L	264	180.1 to 347.8	Yes	****	<0.0001	B-E
F 1μM+L vs. F 2.5μM+L	63.06	-20.78 to 146.9	No	ns	0.2177	C-D
F 1μM+L vs. F 5μM+L	84.44	0.5934 to 168.3	Yes	*	0.0477	C-E
F 2.5μM+L vs. F 5μM+L	21.37	-62.47 to 105.2	No	ns	0.9473	D-E

Figure 1g RT-PCR: cox-2

Number of families	1								
Number of comparisons per family	3								
Alpha	0.05								
Tukey's multiple comparisons test	Mean Diff.	95.00% CI of diff.	Significant?	Summary	Adjusted P Value				
V vs. L	-82.64	-115.9 to -49.34	Yes	****	<0.0001	A-B			
V vs. F+L	-13.91	-47.21 to 19.39	No	ns	0.5729	A-C			
L vs. F+L	68.73	35.43 to 102	Yes	****	<0.0001	B-C			
Test details	Mean 1	Mean 2	Mean Diff.	SE of diff.	n1	n2	q	DF	
V vs. L	100	182.6	-82.64	13.74	16	16	8.507	45	
V vs. F+L	100	113.9	-13.91	13.74	16	16	1.432	45	
L vs. F+L	182.6	113.9	68.73	13.74	16	16	7.075	45	

Figure 1g RT-PCR: il-6

Number of comparisons per family	3								
Alpha	0.05								
Tukey's multiple comparisons test	Mean Diff.	95.00% CI of diff.	Significant?	Summary	Adjusted P Value				
V vs. L	-342.1	-468.6 to -215.6	Yes	****	<0.0001	E-F			
V vs. F+L	-246.3	-372.8 to -119.8	Yes	****	<0.0001	E-G			
L vs. F+L	95.77	-30.71 to 222.3	No	ns	0.1698	F-G			
Test details	Mean 1	Mean 2	Mean Diff.	SE of diff.	n1	n2	q	DF	
V vs. L	100	442.1	-342.1	52.19	16	16	9.271	45	
V vs. F+L	100	346.3	-246.3	52.19	16	16	6.675	45	
L vs. F+L	442.1	346.3	95.77	52.19	16	16	2.595	45	

Figure 1g RT-PCR: il-1β

Number of families	1								
Number of comparisons per family	3								
Alpha	0.05								
Tukey's multiple comparisons test	Mean Diff.	95.00% CI of diff.	Significant?	Summary	Adjusted P Value				
V vs. L	-80.96	-119.1 to -42.83	Yes	****	<0.0001	I-J			
V vs. F+L	-41.63	-79.77 to -3.49	Yes	*	0.0296	I-K			
L vs. F+L	39.34	1.198 to 77.47	Yes	*	0.0419	J-K			
Test details	Mean 1	Mean 2	Mean Diff.	SE of diff.	n1	n2	q	DF	
V vs. L	100	181	-80.96	15.74	16	16	7.276	45	
V vs. F+L	100	141.6	-41.63	15.74	16	16	3.741	45	
L vs. F+L	181	141.6	39.34	15.74	16	16	3.535	45	

Figure 1g RT-PCR: inos

Number of families	1								
Number of comparisons per family	3								
Alpha	0.05								
Tukey's multiple comparisons test	Mean Diff.	95.00% CI of diff.	Significant?	Summary	Adjusted P Value				
V vs. L	-171.1	-253.2 to -88.93	Yes	****	<0.0001	M-N			
V vs. F+L	-95.28	-177.4 to -13.15	Yes	*	0.0195	M-O			
L vs. F+L	75.78	-6.351 to 157.9	No	ns	0.0759	N-O			
Test details	Mean 1	Mean 2	Mean Diff.	SE of diff.	n1	n2	q	DF	
V vs. L	100	271.1	-171.1	33.89	16	16	7.139	45	
V vs. F+L	100	195.3	-95.28	33.89	16	16	3.976	45	
L vs. F+L	271.1	195.3	75.78	33.89	16	16	3.162	45	

Figure 1h ELISA: COX-2

Number of families	1								
Number of comparisons per family	3								
Alpha	0.05								
Tukey's multiple comparisons test	Mean Diff.	95.00% CI of diff.	Significant ?	Summary	Adjusted P Value				
V vs. L	-1891	-3319 to -462.4	Yes	**	0.0099	A-B			
V vs. F+L	-190.5	-1619 to 1238	No	ns	0.9353	A-C			
L vs. F+L	1700	271.9 to 3129	Yes	*	0.0195	B-C			
Test details	Mean 1	Mean 2	Mean Diff.	SE of diff.	n1	n2	q	DF	

V vs. L	1661	3552	-1891	545.8	8	8	4.9	14
V vs. F+L	1661	1851	-190.5	545.8	8	8	0.4937	14
L vs. F+L	3552	1851	1700	545.8	8	8	4.406	14

Figure 1h ELISA: IL-1β

Number of families	1							
Number of comparisons per family	3							
Alpha	0.05							
Tukey's multiple comparisons test	Mean Diff.	95.00% CI of diff.	Significant?	Summary	Adjusted P Value			
V vs. L	-60.92	-139.8 to 17.94	No	ns	0.1505	A-B		
V vs. F+L	76.83	-2.033 to 155.7	No	ns	0.0571	A-C		
L vs. F+L	137.8	58.89 to 216.6	Yes	***	0.0007	B-C		
Test details	Mean 1	Mean 2	Mean Diff.	SE of diff.	n1	n2	q	DF
V vs. L	127.9	188.8	-60.92	31.29	8	8	2.754	21
V vs. F+L	127.9	51.08	76.83	31.29	8	8	3.473	21
L vs. F+L	188.8	51.08	137.8	31.29	8	8	6.226	21

Figure 2a Real time PCR: scrambled siRNA-cacnd1

Number of families	1							
Number of comparisons per family	3							
Alpha	0.05							
Tukey's multiple comparisons test	Mean Diff.	95.00% CI of diff.	Significant?	Summary	Adjusted P Value			
C vs. L	0.1244	-1.596 to 1.845	No	ns	0.9819	A-B		
C vs. 1 μM F+L	-0.2931	-2.014 to 1.428	No	ns	0.9039	A-C		
L vs. 1 μM F+L	-0.4175	-2.138 to 1.303	No	ns	0.8155	B-C		
Test details	Mean 1	Mean 2	Mean Diff.	SE of diff.	n1	n2	q	DF
C vs. L	1	0.8756	0.1244	0.6827	8	8	0.2577	21
C vs. 1 μM F+L	1	1.293	-0.2931	0.6827	8	8	0.6071	21
L vs. 1 μM F+L	0.8756	1.293	-0.4175	0.6827	8	8	0.8648	21

Figure 2a Real time PCR: cacna1d siRNA-cacnd1

Number of families	1							
Number of comparisons per family	3							
Alpha	0.05							
Tukey's multiple comparisons test	Mean Diff.	95.00% CI of diff.	Significant?	Summary	Adjusted P Value			
C vs. L	-0.06589	-0.2113 to 0.07955	No	ns	0.4998	E-F		
C vs. 1 μM F+L	-0.01204	-0.1575 to 0.1334	No	ns	0.9763	E-G		
L vs. 1 μM F+L	0.05385	-0.09159 to 0.1993	No	ns	0.6258	F-G		
Test details	Mean 1	Mean 2	Mean Diff.	SE of diff.	n1	n2	q	DF
C vs. L	0.07868	0.1446	-0.06589	0.0577	8	8	1.615	21
C vs. 1 μM F+L	0.07868	0.09072	-0.01204	0.0577	8	8	0.2951	21
L vs. 1 μM F+L	0.1446	0.09072	0.05385	0.0577	8	8	1.32	21

Figure 2b Real time PCR: scrambled siRNA cox-2

Number of families	1							
Number of comparisons per family	3							
Alpha	0.05							
Tukey's multiple comparisons test	Mean Diff.	95.00% CI of diff.	Significant?	Summary	Adjusted P Value			
C vs. L	-3.78	-7.323 to -0.238	Yes	*	0.0351	A-B		
C vs. 1 μM F+L	0.3308	-3.212 to 3.873	No	ns	0.9700	A-C		
L vs. 1 μM F+L	4.111	0.5688 to 7.653	Yes	*	0.0212	B-C		
Test details	Mean 1	Mean 2	Mean Diff.	SE of diff.	n1	n2	q	DF
C vs. L	1	4.78	-3.78	1.405	8	8	3.804	21
C vs. 1 μM F+L	1	0.6692	0.3308	1.405	8	8	0.3329	21
L vs. 1 μM F+L	4.78	0.6692	4.111	1.405	8	8	4.137	21

Figure 2b Real time PCR: cacna1d siRNA cox-2

Number of families	1

Number of comparisons per family	3							
Alpha	0.05							
Tukey's multiple comparisons test	Mean Diff.	95.00% CI of diff.	Significant ?	Summary	Adjusted P Value			
C vs. L	-2.38	-4.453 to -0.3061	Yes	*	0.0227	E-F		
C vs. 1 µM F+L	-1.044	-3.118 to 1.029	No	ns	0.4273	E-G		
L vs. 1 µM F+L	1.335	-0.7382 to 3.409	No	ns	0.2582	F-G		
Test details	Mean 1	Mean 2	Mean Diff.	SE of diff.	n1	n2	q	DF
C vs. L	0.2741	2.654	-2.38	0.8226	8	8	4.091	21
C vs. 1 µM F+L	0.2741	1.318	-1.044	0.8226	8	8	1.795	21
L vs. 1 µM F+L	2.654	1.318	1.335	0.8226	8	8	2.296	21

Figure 2c Real time PCR: scrambled siRNA il-1β

Number of families	1							
Number of comparisons per family	3							
Alpha	0.05							
Tukey's multiple comparisons test	Mean Diff.	95.00% CI of diff.	Significant ?	Summary	Adjusted P Value			
C vs. L	-8.545	-11.79 to -5.299	Yes	****	<0.0001	A-B		
C vs. 1 µM F+L	0.2752	-2.971 to 3.521	No	ns	0.9752	A-C		
L vs. 1 µM F+L	8.82	5.574 to 12.07	Yes	****	<0.0001	B-C		
Test details	Mean 1	Mean 2	Mean Diff.	SE of diff.	n1	n2	q	DF
C vs. L	1	9.545	-8.545	1.288	8	8	9.383	21
C vs. 1 µM F+L	1	0.7248	0.2752	1.288	8	8	0.3022	21
L vs. 1 µM F+L	9.545	0.7248	8.82	1.288	8	8	9.685	21

Figure 2c Real time PCR: cacna1d siRNA il-1β

Number of families	1							
Number of comparisons per family	3							
Alpha	0.05							
Tukey's multiple comparisons test	Mean Diff.	95.00% CI of diff.	Significant ?	Summary	Adjusted P Value			
C vs. L	-11.02	-17.75 to -4.292	Yes	**	0.0013	E-F		
C vs. 1 µM F+L	-6.321	-13.05 to 0.4056	No	ns	0.0680	E-G		
L vs. 1 µM F+L	4.697	-2.029 to 11.42	No	ns	0.2072	F-G		
Test details	Mean 1	Mean 2	Mean Diff.	SE of diff.	n1	n2	q	DF
C vs. L	0.05988	11.08	-11.02	2.669	8	8	5.839	21
C vs. 1 µM F+L	0.05988	6.381	-6.321	2.669	8	8	3.35	21
L vs. 1 µM F+L	11.08	6.381	4.697	2.669	8	8	2.489	21

Figure 2d Real time PCR: scrambled siRNA-cacnd1

Number of families	1							
Number of comparisons per family	3							
Alpha	0.05							
Tukey's multiple comparisons test	Mean Diff.	95.00% CI of diff.	Significant ?	Summary	Adjusted P Value			
C vs. L	-0.1605	-0.5599 to 0.2389	No	ns	0.5771	A-B		
C vs. 5 µM F+L	-0.1321	-0.5315 to 0.2673	No	ns	0.6867	A-C		
L vs. 5 µM F+L	0.02839	-0.371 to 0.4278	No	ns	0.9825	B-C		
Test details	Mean 1	Mean 2	Mean Diff.	SE of diff.	n1	n2	q	DF
C vs. L	1	1.161	-0.1605	0.1585	8	8	1.433	21
C vs. 5 µM F+L	1	1.132	-0.1321	0.1585	8	8	1.179	21
L vs. 5 µM F+L	1.161	1.132	0.02839	0.1585	8	8	0.2533	21

Figure 2d Real time PCR: cacna1d siRNA-cacnd1

Number of families	1					
Number of comparisons per family	3					
Alpha	0.05					
Tukey's multiple comparisons test	Mean Diff.	95.00% CI of diff.	Significant ?	Summary	Adjusted P Value	
C vs. L	0.01785	-0.05481 to 0.09051	No	ns	0.8114	E-F

C vs. 5 μM F+L	-0.1016	-0.1742 to -0.02889	Yes	**	0.0055	E-G		
L vs. 5 μM F+L	-0.1194	-0.1921 to -0.04674	Yes	**	0.0013	F-G		
Test details	Mean 1	Mean 2	Mean Diff.	SE of diff.	n1	n2	q	DF
C vs. L	0.286	0.2682	0.01785	0.02883	8	8	0.8755	21
C vs. 5 μM F+L	0.286	0.3876	-0.1016	0.02883	8	8	4.982	21
L vs. 5 μM F+L	0.2682	0.3876	-0.1194	0.02883	8	8	5.857	21

Figure 2e Real time PCR: scrambled siRNA cox-2

Number of families	1							
Number of comparisons per family	3							
Alpha	0.05							
Tukey's multiple comparisons test	Mean Diff.	95.00% CI of diff.	Significant ?	Summary	Adjusted P Value			
C vs. L	-6.707	-9.779 to -3.635	Yes	****	<0.0001	A-B		
C vs. 5 μM F+L	-0.1166	-3.189 to 2.956	No	ns	0.9950	A-C		
L vs. 5 μM F+L	6.59	3.518 to 9.662	Yes	****	<0.0001	B-C		
Test details	Mean 1	Mean 2	Mean Diff.	SE of diff.	n1	n2	q	DF
C vs. L	1	7.707	-6.707	1.219	8	8	7.782	21
C vs. 5 μM F+L	1	1.117	-0.1166	1.219	8	8	0.1353	21
L vs. 5 μM F+L	7.707	1.117	6.59	1.219	8	8	7.646	21

Figure 2e Real time PCR: cacna1d siRNA cox-2

Number of families	1							
Number of comparisons per family	3							
Alpha	0.05							
Tukey's multiple comparisons test	Mean Diff.	95.00% CI of diff.	Significant ?	Summary	Adjusted P Value			
C vs. L	-16.17	-20.5 to -11.85	Yes	****	<0.0001	E-F		
C vs. 5 μM F+L	-5.09	-9.416 to -0.7636	Yes	*	0.0194	E-G		
L vs. 5 μM F+L	11.08	6.756 to 15.41	Yes	****	<0.0001	F-G		
Test details	Mean 1	Mean 2	Mean Diff.	SE of diff.	n1	n2	q	DF
C vs. L	0.5483	16.72	-16.17	1.716	8	8	13.33	21
C vs. 5 μM F+L	0.5483	5.638	-5.09	1.716	8	8	4.194	21
L vs. 5 μM F+L	16.72	5.638	11.08	1.716	8	8	9.132	21

Figure 2f Real time PCR: scrambled siRNA il-1β

Number of families	1							
Number of comparisons per family	3							
Alpha	0.05							
Tukey's multiple comparisons test	Mean Diff.	95.00% CI of diff.	Significant ?	Summary	Adjusted P Value			
C vs. L	-78.29	-116.3 to -40.27	Yes	***	0.0001	A-B		
C vs. 5 μM F+L	-5.4	-43.42 to 32.62	No	ns	0.9320	A-C		
L vs. 5 μM F+L	72.89	34.87 to 110.9	Yes	***	0.0003	B-C		
Test details	Mean 1	Mean 2	Mean Diff.	SE of diff.	n1	n2	q	DF
C vs. L	1	79.29	-78.29	15.08	8	8	7.34	21
C vs. 5 μM F+L	1	6.4	-5.4	15.08	8	8	0.5063	21
L vs. 5 μM F+L	79.29	6.4	72.89	15.08	8	8	6.834	21

Tukey's multiple comparisons test	Mean Diff.	95.00% CI of diff.	Significant?	Summary	Adjusted P Value	
5 μM F+L vs. 5 μM F+L	-4.521	-8.965 to -0.0778	Yes	*	0.0440	C-G

Figure 2f Real time PCR: cacna1d siRNA il-1β

Number of families	1							
Number of comparisons per family	3							
Alpha	0.05							
Tukey's multiple comparisons test	Mean Diff.	95.00% CI of diff.	Significant ?	Summary	Adjusted P Value			
C vs. L	-155.4	-217.1 to -93.75	Yes	****	<0.0001	E-F		
C vs. 5 μM F+L	-34.02	-95.69 to 27.66	No	ns	0.3639	E-G		
L vs. 5 μM F+L	121.4	59.73 to 183.1	Yes	***	0.0002	F-G		
Test details	Mean 1	Mean 2	Mean Diff.	SE of diff.	n1	n2	q	DF

C vs. L	1.018	156.4	-155.4	24.47	8	8	8.983	21
C vs. 5 μM F+L	1.018	35.03	-34.02	24.47	8	8	1.966	21
L vs. 5 μM F+L	156.4	35.03	121.4	24.47	8	8	7.017	21

Tukey's multiple comparisons test	Mean Diff.	95.00% CI of diff.	Significant?	Summary	Adjusted P Value	
5 μM F+L vs. 5 μM F+L	-28.63	-43.73 to -13.53	Yes	**	0.0015	C-G

Figure 29 RT-PCR: cox-2

Number of families	1							
Number of comparisons per family	10							
Alpha	0.05							
Newman-Keuls multiple comparisons test	Mean Diff.	Significant ?	Summary					
V vs. L	-18.48	Yes	*		A-B			
V vs. F+L	4.827	No	ns		A-C			
V vs. T+L	2.187	No	ns		A-D			
V vs. T+F+L	12.7	No	ns		A-E			
L vs. F+L	23.31	Yes	*		B-C			
L vs. T+L	20.67	Yes	*		B-D			
L vs. T+F+L	31.18	Yes	**		B-E			
F+L vs. T+L	-2.64	No	ns		C-D			
F+L vs. T+F+L	7.877	No	ns		C-E			
T+L vs. T+F+L	10.52	No	ns		D-E			
Test details	Mean 1	Mean 2	Mean Diff.	SE of diff.	n1	n2	q	DF
V vs. L	100	118.5	-18.48	8.286	22	22	3.154	84
V vs. F+L	100	95.17	4.827	8.286	22	22	---	84
V vs. T+L	100	97.81	2.187	8.286	22	22	---	84
V vs. T+F+L	100	87.3	12.7	8.286	22	22	2.168	84
L vs. F+L	118.5	95.17	23.31	8.286	22	22	3.978	84
L vs. T+L	118.5	97.81	20.67	8.286	22	22	3.527	84
L vs. T+F+L	118.5	87.3	31.18	8.286	22	22	5.322	84
F+L vs. T+L	95.17	97.81	-2.64	8.286	22	22	---	84
F+L vs. T+F+L	95.17	87.3	7.877	8.286	22	22	---	84
T+L vs. T+F+L	97.81	87.3	10.52	8.286	22	22	---	84

Figure 2h Real-time PCR: cox-2

Number of families	1							
Number of comparisons per family	10							
Alpha	0.05							
Tukey's multiple comparisons test	Mean Diff.	95.00% CI of diff.	Significant ?	Summary	Adjusted P Value			
V vs. L	-12.22	-13.68 to -10.76	Yes	****	<0.0001	A-B		
V vs. F+L	0.009866	-1.451 to 1.47	No	ns	>0.9999	A-C		
V vs. T+L	0.2899	-1.171 to 1.75	No	ns	0.9785	A-D		
V vs. T+F+L	0.6711	-0.7894 to 2.132	No	ns	0.6802	A-E		
L vs. F+L	12.23	10.77 to 13.69	Yes	****	<0.0001	B-C		
L vs. T+L	12.51	11.05 to 13.97	Yes	****	<0.0001	B-D		
L vs. T+F+L	12.89	11.43 to 14.35	Yes	****	<0.0001	B-E		
F+L vs. T+L	0.28	-1.18 to 1.741	No	ns	0.9811	C-D		
F+L vs. T+F+L	0.6612	-0.7993 to 2.122	No	ns	0.6920	C-E		
T+L vs. T+F+L	0.3812	-1.079 to 1.842	No	ns	0.9428	D-E		
Test details	Mean 1	Mean 2	Mean Diff.	SE of diff.	n1	n2	q	DF
V vs. L	1	13.22	-12.22	0.508	8	8	34.03	35
V vs. F+L	1	0.9901	0.009866	0.508	8	8	0.02747	35
V vs. T+L	1	0.7101	0.2899	0.508	8	8	0.8071	35
V vs. T+F+L	1	0.3289	0.6711	0.508	8	8	1.868	35
L vs. F+L	13.22	0.9901	12.23	0.508	8	8	34.06	35
L vs. T+L	13.22	0.7101	12.51	0.508	8	8	34.84	35
L vs. T+F+L	13.22	0.3289	12.89	0.508	8	8	35.9	35
F+L vs. T+L	0.9901	0.7101	0.28	0.508	8	8	0.7796	35
F+L vs. T+F+L	0.9901	0.3289	0.6612	0.508	8	8	1.841	35
T+L vs. T+F+L	0.7101	0.3289	0.3812	0.508	8	8	1.061	35

Figure 3a Western blot: p-AKT^{S473}

Number of families	1

Number of comparisons per family	3							
Alpha	0.05							
Tukey's multiple comparisons test	Mean Diff.	95.00% CI of diff.	Significant ?	Summary	Adjusted P Value			
V vs. L	-39.4	-61.4 to -17.4	Yes	**	0.0016	A-B		
V vs. F+L	4.407	-17.6 to 26.41	No	ns	0.8493	A-C		
L vs. F+L	43.8	21.8 to 65.81	Yes	***	0.0007	B-C		
Test details	Mean 1	Mean 2	Mean Diff.	SE of diff.	n1	n2	q	DF
V vs. L	100	139.4	-39.4	8.026	6	6	6.942	10
V vs. F+L	100	95.59	4.407	8.026	6	6	0.7765	10
L vs. F+L	139.4	95.59	43.8	8.026	6	6	7.718	10

Figure 3b Immunocytochemistry: p-AKT^{S473}

Number of families	1							
Number of comparisons per family	3							
Alpha	0.05							
Tukey's multiple comparisons test	Mean Diff.	95.00% CI of diff.	Significant ?	Summary	Adjusted P Value			
V vs. L	-59.45	-69.95 to -48.95	Yes	****	<0.0001	A-B		
V vs. F+L	-0.4024	-10.02 to 9.218	No	ns	0.9947	A-C		
L vs. F+L	59.05	50.22 to 67.88	Yes	****	<0.0001	B-C		
Test details	Mean 1	Mean 2	Mean Diff.	SE of diff.	n1	n2	q	DF
V vs. L	100	159.4	-59.45	4.477	443	578	18.78	1936
V vs. F+L	100	100.4	-0.4024	4.102	443	918	0.1387	1936
L vs. F+L	159.4	100.4	59.05	3.765	578	918	22.18	1936

Figure 3c RT-PCR: cox-2

Number of families	1							
Number of comparisons per family	10							
Alpha	0.05							
Newman-Keuls multiple comparisons test	Mean Diff.	Significant ?	Summary					
V vs. L	-198.7	Yes	**		A-B			
V vs. F+L	-72.29	No	ns		A-C			
V vs. M+L	-11.48	No	ns		A-D			
V vs. M+F+L	32.64	No	ns		A-E			
L vs. F+L	126.4	Yes	**		B-C			
L vs. M+L	187.2	Yes	**		B-D			
L vs. M+F+L	231.3	Yes	***		B-E			
F+L vs. M+L	60.81	No	ns		C-D			
F+L vs. M+F+L	104.9	No	ns		C-E			
M+L vs. M+F+L	44.11	No	ns		D-E			
Test details	Mean 1	Mean 2	Mean Diff.	SE of diff.	n1	n2	q	DF
V vs. L	100	298.7	-198.7	40.48	4	4	6.94	12
V vs. F+L	100	172.3	-72.29	40.48	4	4	---	12
V vs. M+L	100	111.5	-11.48	40.48	4	4	---	12
V vs. M+F+L	100	67.36	32.64	40.48	4	4	---	12
L vs. F+L	298.7	172.3	126.4	40.48	4	4	4.415	12
L vs. M+L	298.7	111.5	187.2	40.48	4	4	6.539	12
L vs. M+F+L	298.7	67.36	231.3	40.48	4	4	8.08	12
F+L vs. M+L	172.3	111.5	60.81	40.48	4	4	---	12
F+L vs. M+F+L	172.3	67.36	104.9	40.48	4	4	3.666	12
M+L vs. M+F+L	111.5	67.36	44.11	40.48	4	4	---	12

Figure 3d Real-time PCR: cox-2

Number of families	1							
Number of comparisons per family	10							
Alpha	0.05							
Tukey's multiple comparisons test	Mean Diff.	95.00% CI of diff.	Significant ?	Summary	Adjusted P Value			
V vs. L	-19.67	-24.16 to -15.18	Yes	****	<0.0001	A-B		
V vs. F+L	-0.682	-5.17 to 3.806	No	ns	0.9916	A-C		
V vs. M+L	0.2521	-4.236 to 4.74	No	ns	0.9998	A-D		
V vs. M+F+L	0.3518	-4.136 to 4.84	No	ns	0.9994	A-E		

L vs. F+L	18.99	14.5 to 23.48	Yes	****	<0.0001	B-C			
L vs. M+L	19.92	15.44 to 24.41	Yes	****	<0.0001	B-D			
L vs. M+F+L	20.02	15.54 to 24.51	Yes	****	<0.0001	B-E			
F+L vs. M+L	0.9341	-3.554 to 5.422	No	ns	0.9729	C-D			
F+L vs. M+F+L	1.034	-3.454 to 5.522	No	ns	0.9610	C-E			
M+L vs. M+F+L	0.09976	-4.388 to 4.588	No	ns	>0.9999	D-E			
Test details	Mean 1	Mean 2	Mean Diff.	SE of diff.	n1	n2	q		DF
V vs. L	1	20.67	-19.67	1.54	8	8	18.06		28
V vs. F+L	1	1.682	-0.682	1.54	8	8	0.6261		28
V vs. M+L	1	0.7479	0.2521	1.54	8	8	0.2314		28
V vs. M+F+L	1	0.6482	0.3518	1.54	8	8	0.323		28
L vs. F+L	20.67	1.682	18.99	1.54	8	8	17.43		28
L vs. M+L	20.67	0.7479	19.92	1.54	8	8	18.29		28
L vs. M+F+L	20.67	0.6482	20.02	1.54	8	8	18.38		28
F+L vs. M+L	1.682	0.7479	0.9341	1.54	8	8	0.8575		28
F+L vs. M+F+L	1.682	0.6482	1.034	1.54	8	8	0.9491		28
M+L vs. M+F+L	0.7479	0.6482	0.09976	1.54	8	8	0.09159		28

Figure 3e Western blot: p-STAT3^{S727}

Number of families	1							
Number of comparisons per family	3							
Alpha	0.05							
Tukey's multiple comparisons test	Mean Diff.	95.00% CI of diff.	Significant?	Summary	Adjusted P Value			
V vs. L	-86.54	-180.7 to 7.609	Yes	*	0.0439	A-B		
V vs. F+L	40.69	-53.47 to 134.8	No	ns	0.5155	A-C		
L vs. F+L	127.2	33.08 to 221.4	Yes	**	0.0083	B-C		
Test details	Mean 1	Mean 2	Mean Diff.	SE of diff.	n1	n2	q	DF
V vs. L	100	186.5	-86.54	36.25	6	6	3.376	15
V vs. F+L	100	59.31	40.69	36.25	6	6	1.587	15
L vs. F+L	186.5	59.31	127.2	36.25	6	6	4.964	15

Figure 3f Immunocytochemistry: p-STAT3^{S727}

Number of families	1							
Number of comparisons per family	3							
Alpha	0.05							
Tukey's multiple comparisons test	Mean Diff.	95.00% CI of diff.	Significant?	Summary	Adjusted P Value			
V vs. L	-119.5	-141.3 to -97.66	Yes	****	<0.0001	A-B		
V vs. F+L	-59.18	-81.35 to -37.01	Yes	****	<0.0001	A-C		
L vs. F+L	60.29	36.88 to 83.7	Yes	****	<0.0001	B-C		
Test details	Mean 1	Mean 2	Mean Diff.	SE of diff.	n1	n2	q	DF
V vs. L	100	219.5	-119.5	9.295	414	326	18.18	1045
V vs. F+L	100	159.2	-59.18	9.446	414	308	8.861	1045
L vs. F+L	219.5	159.2	60.29	9.975	326	308	8.548	1045

Supplementary Figure S1a 24 h MTT

Number of families	1					
Number of comparisons per family	66					
Alpha	0.05					
Tukey's multiple comparisons test	Mean Diff.	95.00% CI of diff.	Significant?	Summary	Adjusted P Value	
DMSO (0.001%) vs. S1885 0.1 uM	-6.819	-16.28 to 2.644	No	ns	0.4149	A-B
DMSO (0.001%) vs. DMSO (0.01%)	-2.5e-006	-9.462 to 9.462	No	ns	>0.9999	A-C
DMSO (0.001%) vs. S1885 1 uM	-4.493	-13.95 to 4.97	No	ns	0.9133	A-D
DMSO (0.001%) vs. DMSO (0.05%)	-9.167e-006	-9.462 to 9.462	No	ns	>0.9999	A-E
DMSO (0.001%) vs. S1885 5 uM	-4.36	-13.82 to 5.102	No	ns	0.9285	A-F
DMSO (0.001%) vs. DMSO (0.1%)	-1.25e-005	-9.462 to 9.462	No	ns	>0.9999	A-G
DMSO (0.001%) vs. S1885 10 uM	7.187	-2.275 to 16.65	No	ns	0.3326	A-H
DMSO (0.001%) vs. DMSO (0.25%)	6.667e-006	-9.462 to 9.462	No	ns	>0.9999	A-I
DMSO (0.001%) vs. S1885 25 uM	-4.405	-13.87 to 5.057	No	ns	0.9235	A-J
DMSO (0.001%) vs. DMSO (0.5%)	-2.5e-006	-9.462 to 9.462	No	ns	>0.9999	A-K
DMSO (0.001%) vs. S1885 50 uM	21.84	12.38 to 31.3	Yes	****	<0.0001	A-L
S1885 0.1 uM vs. DMSO (0.01%)	6.819	-2.644 to 16.28	No	ns	0.4149	B-C
S1885 0.1 uM vs. S1885 1 uM	2.326	-7.136 to 11.79	No	ns	0.9996	B-D
S1885 0.1 uM vs. DMSO (0.05%)	6.819	-2.644 to 16.28	No	ns	0.4149	B-E
S1885 0.1 uM vs. S1885 5 uM	2.459	-7.003 to 11.92	No	ns	0.9993	B-F
S1885 0.1 uM vs. DMSO (0.1%)	6.819	-2.644 to 16.28	No	ns	0.4149	B-G

S1885 0.1 uM vs. S1885 10 uM	14.01	4.544 to 23.47	Yes	***	0.0002	B-H
S1885 0.1 uM vs. DMSO (0.25%)	6.819	-2.644 to 16.28	No	ns	0.4149	B-I
S1885 0.1 uM vs. S1885 25 uM	2.413	-7.049 to 11.88	No	ns	0.9994	B-J
S1885 0.1 uM vs. DMSO (0.5%)	6.819	-2.644 to 16.28	No	ns	0.4149	B-K
S1885 0.1 uM vs. S1885 50 uM	28.66	19.2 to 38.12	Yes	****	<0.0001	B-L
DMSO (0.01%) vs. S1885 1 uM	-4.493	-13.95 to 4.97	No	ns	0.9133	C-D
DMSO (0.01%) vs. DMSO (0.05%)	-6.667e-006	-9.462 to 9.462	No	ns	>0.9999	C-E
DMSO (0.01%) vs. S1885 5 uM	-4.36	-13.82 to 5.102	No	ns	0.9285	C-F
DMSO (0.01%) vs. DMSO (0.1%)	-1e-005	-9.462 to 9.462	No	ns	>0.9999	C-G
DMSO (0.01%) vs. S1885 10 uM	7.187	-2.275 to 16.65	No	ns	0.3326	C-H
DMSO (0.01%) vs. DMSO (0.25%)	9.167e-006	-9.462 to 9.462	No	ns	>0.9999	C-I
DMSO (0.01%) vs. S1885 25 uM	-4.405	-13.87 to 5.057	No	ns	0.9235	C-J
DMSO (0.01%) vs. DMSO (0.5%)	0	-9.462 to 9.462	No	ns	>0.9999	C-K
DMSO (0.01%) vs. S1885 50 uM	21.84	12.38 to 31.3	Yes	****	<0.0001	C-L
S1885 1 uM vs. DMSO (0.05%)	4.493	-4.97 to 13.95	No	ns	0.9133	D-E
S1885 1 uM vs. S1885 5 uM	0.1327	-9.33 to 9.595	No	ns	>0.9999	D-F
S1885 1 uM vs. DMSO (0.1%)	4.493	-4.97 to 13.95	No	ns	0.9133	D-G
S1885 1 uM vs. S1885 10 uM	11.68	2.217 to 21.14	Yes	**	0.0039	D-H
S1885 1 uM vs. DMSO (0.25%)	4.493	-4.97 to 13.95	No	ns	0.9133	D-I
S1885 1 uM vs. S1885 25 uM	0.08731	-9.375 to 9.55	No	ns	>0.9999	D-J
S1885 1 uM vs. DMSO (0.5%)	4.493	-4.97 to 13.95	No	ns	0.9133	D-K
S1885 1 uM vs. S1885 50 uM	26.34	16.87 to 35.8	Yes	****	<0.0001	D-L
DMSO (0.05%) vs. S1885 5 uM	-4.36	-13.82 to 5.102	No	ns	0.9285	E-F
DMSO (0.05%) vs. DMSO (0.1%)	-3.333e-006	-9.462 to 9.462	No	ns	>0.9999	E-G
DMSO (0.05%) vs. S1885 10 uM	7.187	-2.275 to 16.65	No	ns	0.3326	E-H
DMSO (0.05%) vs. DMSO (0.25%)	1.583e-005	-9.462 to 9.462	No	ns	>0.9999	E-I
DMSO (0.05%) vs. S1885 25 uM	-4.405	-13.87 to 5.057	No	ns	0.9235	E-J
DMSO (0.05%) vs. DMSO (0.5%)	6.667e-006	-9.462 to 9.462	No	ns	>0.9999	E-K
DMSO (0.05%) vs. S1885 50 uM	21.84	12.38 to 31.3	Yes	****	<0.0001	E-L
S1885 5 uM vs. DMSO (0.1%)	4.36	-5.102 to 13.82	No	ns	0.9285	F-G
S1885 5 uM vs. S1885 10 uM	11.55	2.085 to 21.01	Yes	**	0.0046	F-H
S1885 5 uM vs. DMSO (0.25%)	4.36	-5.102 to 13.82	No	ns	0.9285	F-I
S1885 5 uM vs. S1885 25 uM	-0.04536	-9.508 to 9.417	No	ns	>0.9999	F-J
S1885 5 uM vs. DMSO (0.5%)	4.36	-5.102 to 13.82	No	ns	0.9285	F-K
S1885 5 uM vs. S1885 50 uM	26.2	16.74 to 35.66	Yes	****	<0.0001	F-L
DMSO (0.1%) vs. S1885 10 uM	7.187	-2.275 to 16.65	No	ns	0.3326	G-H
DMSO (0.1%) vs. DMSO (0.25%)	1.917e-005	-9.462 to 9.462	No	ns	>0.9999	G-I
DMSO (0.1%) vs. S1885 25 uM	-4.405	-13.87 to 5.057	No	ns	0.9235	G-J
DMSO (0.1%) vs. DMSO (0.5%)	1e-005	-9.462 to 9.462	No	ns	>0.9999	G-K
DMSO (0.1%) vs. S1885 50 uM	21.84	12.38 to 31.3	Yes	****	<0.0001	G-L
S1885 10 uM vs. DMSO (0.25%)	-7.187	-16.65 to 2.275	No	ns	0.3326	H-I
S1885 10 uM vs. S1885 25 uM	-11.59	-21.05 to -2.13	Yes	**	0.0043	H-J
S1885 10 uM vs. DMSO (0.5%)	-7.187	-16.65 to 2.275	No	ns	0.3326	H-K
S1885 10 uM vs. S1885 50 uM	14.66	5.193 to 24.12	Yes	****	<0.0001	H-L
DMSO (0.25%) vs. S1885 25 uM	-4.405	-13.87 to 5.057	No	ns	0.9235	I-J
DMSO (0.25%) vs. DMSO (0.5%)	-9.167e-006	-9.462 to 9.462	No	ns	>0.9999	I-K
DMSO (0.25%) vs. S1885 50 uM	21.84	12.38 to 31.3	Yes	****	<0.0001	I-L
S1885 25 uM vs. DMSO (0.5%)	4.405	-5.057 to 13.87	No	ns	0.9235	J-K
S1885 25 uM vs. S1885 50 uM	26.25	16.79 to 35.71	Yes	****	<0.0001	J-L
DMSO (0.5%) vs. S1885 50 uM	21.84	12.38 to 31.3	Yes	****	<0.0001	K-L

Supplementary Figure S1b RT-PCR: cox-2

Number of families	1
Number of comparisons per family	3
Alpha	0.05

Tukey's multiple comparisons test	Mean Diff.	95.00% CI of diff.	Significant?	Summary	Adjusted P Value	
V vs. L	-42.08	-83.26 to -0.8936	Yes	*	0.0447	A-B
V vs. L+N	-3.814	-45 to 37.37	No	ns	0.9704	A-C
L vs. L+N	38.26	-2.921 to 79.44	No	ns	0.0717	B-C

Test details	Mean 1	Mean 2	Mean Diff.	SE of diff.	n1	n2	q	DF
V vs. L	100	142.1	-42.08	16.34	8	8	3.642	21
V vs. L+N	100	103.8	-3.814	16.34	8	8	0.3301	21
L vs. L+N	142.1	103.8	38.26	16.34	8	8	3.312	21

Supplementary Figure S1b RT-PCR: il-6

Number of families	1

Number of comparisons per family	3							
Alpha	0.05							
Tukey's multiple comparisons test	Mean Diff.	95.00% CI of diff.	Significant?	Summary	Adjusted P Value			
V vs. L	-241.1	-349.3 to -132.8	Yes	****	<0.0001	E-F		
V vs. L+N	-153.6	-261.9 to -45.38	Yes	**	0.0048	E-G		
L vs. L+N	87.45	-20.81 to 195.7	No	ns	0.1282	F-G		
Test details	Mean 1	Mean 2	Mean Diff.	SE of diff.	n1	n2	q	DF
V vs. L	100	341.1	-241.1	42.95	8	8	7.938	21
V vs. L+N	100	253.6	-153.6	42.95	8	8	5.059	21
L vs. L+N	341.1	253.6	87.45	42.95	8	8	2.879	21

Supplementary Figure S1b RT-PCR: il-1β

Number of families	1							
Number of comparisons per family	3							
Alpha	0.05							
Tukey's multiple comparisons test	Mean Diff.	95.00% CI of diff.	Significant?	Summary	Adjusted P Value			
V vs. L	-87.13	-156.7 to -17.56	Yes	*	0.0127	I-J		
V vs. L+N	-17.52	-87.09 to 52.05	No	ns	0.8028	I-K		
L vs. L+N	69.61	0.03612 to 139.2	Yes	*	0.0499	J-K		
Test details	Mean 1	Mean 2	Mean Diff.	SE of diff.	n1	n2	q	DF
V vs. L	100	187.1	-87.13	27.6	8	8	4.464	21
V vs. L+N	100	117.5	-17.52	27.6	8	8	0.8978	21
L vs. L+N	187.1	117.5	69.61	27.6	8	8	3.566	21

Supplementary Figure S1b RT-PCR: inos

Number of families	1							
Number of comparisons per family	3							
Alpha	0.05							
Tukey's multiple comparisons test	Mean Diff.	95.00% CI of diff.	Significant?	Summary	Adjusted P Value			
V vs. L	-38.67	-79.33 to 1.992	No	ns	0.0642	M-N		
V vs. L+N	-1.845	-42.51 to 38.82	No	ns	0.9928	M-O		
L vs. L+N	36.82	-3.836 to 77.49	No	ns	0.0805	N-O		
Test details	Mean 1	Mean 2	Mean Diff.	SE of diff.	n1	n2	q	DF
V vs. L	100	138.7	-38.67	16.13	8	8	3.39	21
V vs. L+N	100	101.8	-1.845	16.13	8	8	0.1617	21
L vs. L+N	138.7	101.8	36.82	16.13	8	8	3.228	21

Supplementary Figure S1c RT-PCR: cox-2

Number of families	1							
Number of comparisons per family	3							
Alpha	0.05							
Tukey's multiple comparisons test	Mean Diff.	95.00% CI of diff.	Significant?	Summary	Adjusted P Value			
V vs. L	-80.68	-116.1 to -45.26	Yes	****	<0.0001	A-B		
V vs. N+L	-25.43	-60.85 to 9.988	No	ns	0.1908	A-C		
L vs. N+L	55.25	19.83 to 90.67	Yes	**	0.0021	B-C		
Test details	Mean 1	Mean 2	Mean Diff.	SE of diff.	n1	n2	q	DF
V vs. L	100	180.7	-80.68	14.05	8	8	8.12	21
V vs. N+L	100	125.4	-25.43	14.05	8	8	2.559	21
L vs. N+L	180.7	125.4	55.25	14.05	8	8	5.561	21

Supplementary Figure S1c RT-PCR: il-6

Number of families	1							
Number of comparisons per family	3							
Alpha	0.05							
Tukey's multiple comparisons test	Mean Diff.	95.00% CI of diff.	Significant?	Summary	Adjusted P Value			
V vs. L	-267.8	-344.3 to -191.3	Yes	****	<0.0001	E-F		
V vs. N+L	-130.4	-206.9 to -53.91	Yes	***	0.0009	E-G		
L vs. N+L	137.4	60.88 to 213.9	Yes	***	0.0005	F-G		
Test details	Mean 1	Mean 2	Mean Diff.	SE of diff.	n1	n2	q	DF

V vs. L	100	367.8	-267.8	30.35	8	8	12.48	21
V vs. N+L	100	230.4	-130.4	30.35	8	8	6.076	21
L vs. N+L	367.8	230.4	137.4	30.35	8	8	6.401	21

Supplementary Figure S1c RT-PCR: il-1β

Number of families	1							
Number of comparisons per family	3							
Alpha	0.05							
Tukey's multiple comparisons test	Mean Diff.	95.00% CI of diff.	Significant?	Summary	Adjusted P Value			
V vs. L	-103.5	-138 to -69	Yes	****	<0.0001	I-J		
V vs. N+L	-32.56	-67.05 to 1.932	No	ns	0.0665	I-K		
L vs. N+L	70.93	36.44 to 105.4	Yes	***	0.0001	J-K		
Test details	Mean 1	Mean 2	Mean Diff.	SE of diff.	n1	n2	q	DF
V vs. L	100	203.5	-103.5	13.68	8	8	10.7	21
V vs. N+L	100	132.6	-32.56	13.68	8	8	3.365	21
L vs. N+L	203.5	132.6	70.93	13.68	8	8	7.331	21

Supplementary Figure S1c RT-PCR: inos

Number of families	1							
Number of comparisons per family	3							
Alpha	0.05							
Tukey's multiple comparisons test	Mean Diff.	95.00% CI of diff.	Significant?	Summary	Adjusted P Value			
V vs. L	-56.42	-100 to -12.84	Yes	**	0.0100	M-N		
V vs. N+L	-22.64	-66.22 to 20.94	No	ns	0.4056	M-O		
L vs. N+L	33.78	-9.802 to 77.36	No	ns	0.1487	N-O		
Test details	Mean 1	Mean 2	Mean Diff.	SE of diff.	n1	n2	q	DF
V vs. L	100	156.4	-56.42	17.29	8	8	4.615	21
V vs. N+L	100	122.6	-22.64	17.29	8	8	1.852	21
L vs. N+L	156.4	122.6	33.78	17.29	8	8	2.763	21

Supplementary Figure S2a Real-time PCR: Scr-SiRNA cacna1d

Number of families	1							
Number of comparisons per family	3							
Alpha	0.05							
Tukey's multiple comparisons test	Mean Diff.	95.00% CI of diff.	Significant?	Summary	Adjusted P Value			
C vs. L	-0.09956	-0.5582 to 0.3591	No	ns	0.8490	A-B		
C vs. N+L	-0.08618	-0.5448 to 0.3725	No	ns	0.8844	A-C		
L vs. N+L	0.01337	-0.4453 to 0.472	No	ns	0.9970	B-C		
Test details	Mean 1	Mean 2	Mean Diff.	SE of diff.	n1	n2	q	DF
C vs. L	1	1.1	-0.09956	0.182	8	8	0.7738	21
C vs. N+L	1	1.086	-0.08618	0.182	8	8	0.6698	21
L vs. N+L	1.1	1.086	0.01337	0.182	8	8	0.1039	21

Supplementary Figure S2a Real-time PCR: cacnd1-SiRNA cacna1d cancnd1

Number of families	1							
Number of comparisons per family	3							
Alpha	0.05							
Tukey's multiple comparisons test	Mean Diff.	95.00% CI of diff.	Significant ?	Summary	Adjusted P Value			
C vs. L	0.005076	-0.1183 to 0.1285	No	ns	0.9941	E-F		
C vs. N+L	-0.08574	-0.2091 to 0.03765	No	ns	0.2103	E-G		
L vs. N+L	-0.09081	-0.2142 to 0.03258	No	ns	0.1766	F-G		
Test details	Mean 1	Mean 2	Mean Diff.	SE of diff.	n1	n2	q	DF
C vs. L	0.2576	0.2525	0.005076	0.04895	8	8	0.1466	21
C vs. N+L	0.2576	0.3433	-0.08574	0.04895	8	8	2.477	21
L vs. N+L	0.2525	0.3433	-0.09081	0.04895	8	8	2.623	21

Supplementary Figure S2b Real-time PCR: Scr-SiRNA cox-2

Number of families	1
Number of comparisons per family	3

Alpha	0.05							
Tukey's multiple comparisons test	Mean Diff.	95.00% CI of diff.	Significant?	Summary	Adjusted P Value			
C vs. L	-20.49	-26.4 to -14.59	Yes	****	<0.0001	A-B		
C vs. N+L	-1.612	-7.32 to 4.096	No	ns	0.7578	A-C		
L vs. N+L	18.88	12.97 to 24.79	Yes	****	<0.0001	B-C		
Test details	Mean 1	Mean 2	Mean Diff.	SE of diff.	n1	n2	q	DF
C vs. L	1	21.49	-20.49	2.335	8	7	12.41	20
C vs. N+L	1	2.612	-1.612	2.256	8	8	1.01	20
L vs. N+L	21.49	2.612	18.88	2.335	7	8	11.44	20

Supplementary Figure S2b Real-time PCR: cacnd1-SiRNA cox-2

Number of families	1							
Number of comparisons per family	3							
Alpha	0.05							
Tukey's multiple comparisons test	Mean Diff.	95.00% CI of diff.	Significant ?	Summary	Adjusted P Value			
C vs. L	-15.26	-20.25 to -10.27	Yes	****	<0.0001	E-F		
C vs. N+L	-6.334	-11.15 to -1.513	Yes	**	0.0091	E-G		
L vs. N+L	8.925	3.935 to 13.91	Yes	***	0.0006	F-G		
Test details	Mean 1	Mean 2	Mean Diff.	SE of diff.	n1	n2	q	DF
C vs. L	1.098	16.36	-15.26	1.972	8	7	10.94	20
C vs. N+L	1.098	7.432	-6.334	1.905	8	8	4.701	20
L vs. N+L	16.36	7.432	8.925	1.972	7	8	6.4	20

Supplementary Figure S2c Real-time PCR: il-1β

Number of families	1							
Number of comparisons per family	3							
Alpha	0.05							
Tukey's multiple comparisons test	Mean Diff.	95.00% CI of diff.	Significant?	Summary	Adjusted P Value			
C vs. L	-156.3	-213.4 to -99.31	Yes	****	<0.0001	A-B		
C vs. N+L	-7.727	-64.76 to 49.31	No	ns	0.9379	A-C		
L vs. N+L	148.6	91.59 to 205.7	Yes	****	<0.0001	B-C		
Test details	Mean 1	Mean 2	Mean Diff.	SE of diff.	n1	n2	q	DF
C vs. L	1	157.3	-156.3	22.63	8	8	9.771	21
C vs. N+L	1	8.727	-7.727	22.63	8	8	0.4829	21
L vs. N+L	157.3	8.727	148.6	22.63	8	8	9.288	21

Supplementary Figure S2c Real-time PCR: il-1β

Number of families	1							
Number of comparisons per family	3							
Alpha	0.05							
Tukey's multiple comparisons test	Mean Diff.	95.00% CI of diff.	Significant ?	Summary	Adjusted P Value			
C vs. L	-111.6	-145.3 to -78.01	Yes	****	<0.0001	E-F		
C vs. N+L	-22.27	-55.91 to 11.37	No	ns	0.2405	E-G		
L vs. N+L	89.38	55.74 to 123	Yes	****	<0.0001	F-G		
Test details	Mean 1	Mean 2	Mean Diff.	SE of diff.	n1	n2	q	DF
C vs. L	1.234	112.9	-111.6	13.35	8	8	11.83	21
C vs. N+L	1.234	23.5	-22.27	13.35	8	8	2.36	21
L vs. N+L	112.9	23.5	89.38	13.35	8	8	9.471	21

Supplementary Figure S3 RT-PCR: cox-2

Number of families	1					
Number of comparisons per family	10					
Alpha	0.05					
Tukey's multiple comparisons test	Mean Diff.	95.00% CI of diff.	Significant ?	Summary	Adjusted P Value	
V vs. L	-154.9	-249.1 to -60.76	Yes	**	0.0016	A-B
V vs. N+L	-39.74	-133.9 to 54.44	No	ns	0.6708	A-C
V vs. T+N	1.978	-92.2 to 96.16	No	ns	>0.9999	A-D
V vs. T+N+L	5.126	-89.05 to 99.31	No	ns	0.9998	A-E
L vs. N+L	115.2	21.02 to 209.4	Yes	*	0.0148	B-C
L vs. T+N	156.9	62.74 to 251.1	Yes	**	0.0014	B-D
L vs. T+N+L	160.1	65.88 to 254.2	Yes	**	0.0012	B-E

N+L vs. T+N	41.71	-52.47 to 135.9	No	ns	0.6322	C-D
N+L vs. T+N+L	44.86	-49.32 to 139	No	ns	0.5707	C-E
T+N vs. T+N+L	3.148	-91.03 to 97.33	No	ns	>0.9999	D-E

Test details	Mean 1	Mean 2	Mean Diff.	SE of diff.	n1	n2	q	DF
V vs. L	100	254.9	-154.9	29.55	4	4	7.416	12
V vs. N+L	100	139.7	-39.74	29.55	4	4	1.902	12
V vs. T+N	100	98.02	1.978	29.55	4	4	0.09468	12
V vs. T+N+L	100	94.87	5.126	29.55	4	4	0.2454	12
L vs. N+L	254.9	139.7	115.2	29.55	4	4	5.514	12
L vs. T+N	254.9	98.02	156.9	29.55	4	4	7.51	12
L vs. T+N+L	254.9	94.87	160.1	29.55	4	4	7.661	12
N+L vs. T+N	139.7	98.02	41.71	29.55	4	4	1.997	12
N+L vs. T+N+L	139.7	94.87	44.86	29.55	4	4	2.147	12
T+N vs. T+N+L	98.02	94.87	3.148	29.55	4	4	0.1507	12

Supplementary Figure S4a Western blot: p-AKT^{S473}

Number of families	1					
Number of comparisons per family	3					
Alpha	0.05					
Tukey's multiple comparisons test	Mean Diff.	95.00% CI of diff.	Significant?	Summary	Adjusted P Value	
V vs. L	-59.1	-94.78 to -23.42	Yes	**	0.0010	A-B
V vs. N+L	-13.88	-49.56 to 21.8	No	ns	0.6016	A-C
L vs. N+L	45.22	9.545 to 80.9	Yes	*	0.0112	B-C
Test details	Mean 1	Mean 2	Mean Diff.	SE of diff.	n1	n2
V vs. L	100	159.1	-59.1	14.29	13	13
V vs. N+L	100	113.9	-13.88	14.29	13	13
L vs. N+L	159.1	113.9	45.22	14.29	13	13

Supplementary Figure S5a Western blot: p-AKT^{Y308}

Number of families	1							
Number of comparisons per family	3							
Alpha	0.05							
Tukey's multiple comparisons test	Mean Diff.	95.00% CI of diff.	Significant ?	Summary	Adjusted P Value			
V vs. L	-52.57	-109.8 to 4.653	Yes	*	0.0429	A-B		
V vs. F+L	-30.23	-87.45 to 26.99	No	ns	0.3671	A-C		
L vs. F+L	22.34	-34.88 to 79.56	No	ns	0.5662	B-C		
Test details	Mean 1	Mean 2	Mean Diff.	SE of diff.	n1	n2	q	DF
V vs. L	100	152.6	-52.57	21.45	7	7	3.466	12
V vs. F+L	100	130.2	-30.23	21.45	7	7	1.993	12
L vs. F+L	152.6	130.2	22.34	21.45	7	7	1.473	12

Supplementary Figure S5b Immunocytochemistry: p-AKT^{Y308}

Number of families	1							
Number of comparisons per family	3							
Alpha	0.05							
Tukey's multiple comparisons test	Mean Diff.	95.00% CI of diff.	Significant ?	Summary	Adjusted P Value			
V vs. L	-37.57	-58.37 to -16.76	Yes	****	<0.0001	A-B		
V vs. F+L	-37.68	-57.17 to -18.19	Yes	****	<0.0001	A-C		
L vs. F+L	-0.1096	-17.91 to 17.7	No	ns	0.9999	B-C		
Test details	Mean 1	Mean 2	Mean Diff.	SE of diff.	n1	n2	q	DF
V vs. L	100	137.6	-37.57	8.858	171	229	5.998	718
V vs. F+L	100	137.7	-37.68	8.298	171	321	6.422	718
L vs. F+L	137.6	137.7	-0.1096	7.581	229	321	0.02045	718

Supplementary Figure S6a Western blot: p-ERK

Number of families	1					
Number of comparisons per family	3					
Alpha	0.05					
Tukey's multiple comparisons test	Mean Diff.	95.00% CI of diff.	Significant ?	Summary	Adjusted P Value	
V vs. L	-256.6	-341.2 to -172.1	Yes	****	<0.0001	A-B

V vs. F+L	-249.4	-334 to -164.9	Yes	****	<0.0001	A-C			
L vs. F+L	7.18	-77.36 to 91.72	No	ns	0.9736	B-C			
Test details	Mean 1	Mean 2	Mean Diff.	SE of diff.	n1	n2	q	DF	
V vs. L	100	356.6	-256.6	32.55	6	6	11.15	15	
V vs. F+L	100	349.4	-249.4	32.55	6	6	10.84	15	
L vs. F+L	356.6	349.4	7.18	32.55	6	6	0.312	15	
**Supplementary Figure S6b Western blot: p-p38**									
Number of families	1								
Number of comparisons per family	3								
Alpha	0.05								
Tukey's multiple comparisons test	Mean Diff.	95.00% CI of diff.	Significant ?	Summary	Adjusted P Value				
V vs. L	-143.2	-345.4 to 59.08	No	ns	0.1910	A-B			
V vs. F+L	-154.2	-356.5 to 48.05	No	ns	0.1513	A-C			
L vs. F+L	-11.03	-213.3 to 191.2	No	ns	0.9890	B-C			
Test details	Mean 1	Mean 2	Mean Diff.	SE of diff.	n1	n2	q	DF	
V vs. L	100	243.2	-143.2	77.87	6	6	2.6	15	
V vs. F+L	100	254.2	-154.2	77.87	6	6	2.801	15	
L vs. F+L	243.2	254.2	-11.03	77.87	6	6	0.2003	15	
**Supplementary Figure S7 Immunocytochemistry: p-STAT3^{S727}**									
Number of families	1								
Number of comparisons per family	3								
Alpha	0.05								
Tukey's multiple comparisons test	Mean Diff.	95.00% CI of diff.	Significant ?	Summary	Adjusted P Value				
V vs. L	-87.59	-99 to -76.17	Yes	****	<0.0001	A-B			
V vs. N+L	34.37	22.69 to 46.05	Yes	****	<0.0001	A-C			
L vs. N+L	122	111.4 to 132.5	Yes	****	<0.0001	B-C			
Test details	Mean 1	Mean 2	Mean Diff.	SE of diff.	n1	n2	q	DF	
V vs. L	100	187.6	-87.59	4.867	392	581	25.45	1492	
V vs. N+L	100	65.63	34.37	4.977	392	522	9.767	1492	
L vs. N+L	187.6	65.63	122	4.491	581	522	38.41	1492	
**Supplementary Figure S8 Immunocytochemistry: p-NF-kB^{S536}**									
Number of families	1								
Number of comparisons per family	3								
Alpha	0.05								
Tukey's multiple comparisons test	Mean Diff.	95.00% CI of diff.	Significant ?	Summary	Adjusted P Value				
V vs. L	-20.63	-29.44 to -11.83	Yes	****	<0.0001	A-B			
V vs. F+L	-12.56	-22.58 to -2.538	Yes	**	0.0094	A-C			
L vs. F+L	8.074	-1.349 to 17.5	No	ns	0.1100	B-C			
Test details	Mean 1	Mean 2	Mean Diff.	SE of diff.	n1	n2	q	DF	
V vs. L	100	120.6	-20.63	3.75	267	361	7.782	838	
V vs. F+L	100	112.6	-12.56	4.268	267	213	4.161	838	
L vs. F+L	120.6	112.6	8.074	4.014	361	213	2.845	838	

Supplementary Table S2. One-way ANOVA (Tukey's test) and significance of the results of the *in vivo* experiments in this study.

Figure 4d Immunofluorescence staining: Iba-1 fluorescence intensity-Cortex									
Number of families	1								
Number of comparisons per family	3								
Alpha	0.05								
Tukey's multiple comparisons test	Mean Diff.	95.00% CI of diff.	Significant ?	Summary	Adjusted P Value				
V vs. L	-81.63	-121 to -42.29	Yes	****	<0.0001	A-B			
V vs. F+L	-3.808	-45.54 to 37.92	No	ns	0.9737	A-C			
L vs. F+L	77.82	36.09 to 119.6	Yes	***	0.0001	B-C			
Test details	Mean 1	Mean 2	Mean Diff.	SE of diff.	n1	n2	q	DF	
V vs. L	100	181.6	-81.63	16.32	20	20	7.075	53	
V vs. F+L	100	103.8	-3.808	17.31	20	16	0.3112	53	
L vs. F+L	181.6	103.8	77.82	17.31	20	16	6.36	53	
**Figure 4d Immunofluorescence staining: Iba-1 fluorescence intensity-CA1**									
Number of families	1								
Number of comparisons per family	3								
Alpha	0.05								
Tukey's multiple comparisons test	Mean Diff.	95.00% CI of diff.	Significant ?	Summary	Adjusted P Value				
V vs. L	-89.72	-142.4 to -37.07	Yes	***	0.0004	E-F			
V vs. F+L	-67.4	-122.3 to -12.49	Yes	*	0.0125	E-G			
L vs. F+L	22.31	-32.6 to 77.23	No	ns	0.5930	F-G			
Test details	Mean 1	Mean 2	Mean Diff.	SE of diff.	n1	n2	q	DF	
V vs. L	100	189.7	-89.72	21.84	20	20	5.809	54	
V vs. F+L	100	167.4	-67.4	22.79	20	17	4.183	54	
L vs. F+L	189.7	167.4	22.31	22.79	20	17	1.385	54	
**Figure 4d Immunofluorescence staining: Iba-1 fluorescence intensity-DG**									
Number of families	1								
Number of comparisons per family	3								
Alpha	0.05								
Tukey's multiple comparisons test	Mean Diff.	95.00% CI of diff.	Significant ?	Summary	Adjusted P Value				
V vs. L	-76.11	-124.6 to -27.65	Yes	**	0.0011	I-J			
V vs. F+L	-57.78	-108.9 to -6.608	Yes	*	0.0233	I-K			
L vs. F+L	18.33	-32.27 to 68.94	No	ns	0.6597	J-K			
Test details	Mean 1	Mean 2	Mean Diff.	SE of diff.	n1	n2	q	DF	
V vs. L	100	176.1	-76.11	20.12	20	21	5.35	55	
V vs. F+L	100	157.8	-57.78	21.24	20	17	3.846	55	
L vs. F+L	176.1	157.8	18.33	21.01	21	17	1.234	55	
**Figure 4d Immunofluorescence staining: Iba-1 fluorescence intensity-CA3**									
Number of families	1								
Number of comparisons per family	3								
Alpha	0.05								
Tukey's multiple comparisons test	Mean Diff.	95.00% CI of diff.	Significant ?	Summary	Adjusted P Value				
V vs. L	-35.45	-86.02 to 15.13	No	ns	0.2186	M-N			
V vs. F+L	-31.65	-84.41 to 21.11	No	ns	0.3251	M-O			
L vs. F+L	3.797	-48.96 to 56.55	No	ns	0.9836	N-O			
Test details	Mean 1	Mean 2	Mean Diff.	SE of diff.	n1	n2	q	DF	
V vs. L	100	135.4	-35.45	20.98	20	20	2.389	54	
V vs. F+L	100	131.6	-31.65	21.89	20	17	2.045	54	
L vs. F+L	135.4	131.6	3.797	21.89	20	17	0.2453	54	
**Figure 4d Immunofluorescence staining: Iba-1 (+) cells/mm^2-Cortex**									
Number of families	1								
Number of comparisons per family	3								

Alpha	0.05							
Tukey's multiple comparisons test	Mean Diff.	95.00% CI of diff.	Significant ?	Summary	Adjusted P Value			
V vs. L	-99.8	-131.7 to -67.91	Yes	****	<0.0001	A-B		
V vs. F+L	0.572	-32.7 to 33.85	No	ns	0.9991	A-C		
L vs. F+L	100.4	67.1 to 133.7	Yes	****	<0.0001	B-C		
Test details	Mean 1	Mean 2	Mean Diff.	SE of diff.	n1	n2	q	DF
V vs. L	157.6	257.4	-99.8	13.24	20	20	10.66	54
V vs. F+L	157.6	157	0.572	13.81	20	17	0.05859	54
L vs. F+L	257.4	157	100.4	13.81	20	17	10.28	54

Figure 4d Immunofluorescence staining: Iba-1 (+) cells/mm^2-CA1

Number of families	1							
Number of comparisons per family	3							
Alpha	0.05							
Tukey's multiple comparisons test	Mean Diff.	95.00% CI of diff.	Significant ?	Summary	Adjusted P Value			
V vs. L	-60.16	-115.2 to -5.082	Yes	*	0.0292	E-F		
V vs. F+L	18.8	-38.65 to 76.26	No	ns	0.7115	E-G		
L vs. F+L	78.96	21.51 to 136.4	Yes	**	0.0046	F-G		
Test details	Mean 1	Mean 2	Mean Diff.	SE of diff.	n1	n2	q	DF
V vs. L	190.6	250.7	-60.16	22.85	20	20	3.723	54
V vs. F+L	190.6	171.8	18.8	23.84	20	17	1.115	54
L vs. F+L	250.7	171.8	78.96	23.84	20	17	4.684	54

Figure 4d Immunofluorescence staining: Iba-1 (+) cells/mm^2-DG

Number of families	1							
Number of comparisons per family	3							
Alpha	0.05							
Tukey's multiple comparisons test	Mean Diff.	95.00% CI of diff.	Significant ?	Summary	Adjusted P Value			
V vs. L	-57.99	-99.18 to -16.81	Yes	**	0.0037	I-J		
V vs. F+L	-3.296	-46.26 to 39.67	No	ns	0.9813	I-K		
L vs. F+L	54.7	11.73 to 97.66	Yes	**	0.0093	J-K		
Test details	Mean 1	Mean 2	Mean Diff.	SE of diff.	n1	n2	q	DF
V vs. L	178.9	236.9	-57.99	17.09	20	20	4.799	54
V vs. F+L	178.9	182.2	-3.296	17.83	20	17	0.2615	54
L vs. F+L	236.9	182.2	54.7	17.83	20	17	4.339	54

Figure 4d Immunofluorescence staining: Iba-1 (+) cells/mm^2-CA3

Number of families	1							
Number of comparisons per family	3							
Alpha	0.05							
Tukey's multiple comparisons test	Mean Diff.	95.00% CI of diff.	Significant ?	Summary	Adjusted P Value			
V vs. L	-22.66	-73.43 to 28.11	No	ns	0.5332	M-N		
V vs. F+L	19.68	-33.28 to 72.64	No	ns	0.6454	M-O		
L vs. F+L	42.34	-10.62 to 95.3	No	ns	0.1410	N-O		
Test details	Mean 1	Mean 2	Mean Diff.	SE of diff.	n1	n2	q	DF
V vs. L	111.8	134.4	-22.66	21.07	20	20	1.521	54
V vs. F+L	111.8	92.07	19.68	21.98	20	17	1.267	54
L vs. F+L	134.4	92.07	42.34	21.98	20	17	2.725	54

Figure 4d Immunofluorescence staining: Iba-1 area fraction %-Cortex

Number of families	1							
Number of comparisons per family	3							
Alpha	0.05							
Tukey's multiple comparisons test	Mean Diff.	95.00% CI of diff.	Significant ?	Summary	Adjusted P Value			
V vs. L	-0.4731	-0.6401 to -0.3061	Yes	****	<0.0001	A-B		
V vs. F+L	-0.05103	-0.2282 to 0.1261	No	ns	0.7678	A-C		
L vs. F+L	0.4221	0.2449 to 0.5992	Yes	****	<0.0001	B-C		
Test details	Mean 1	Mean 2	Mean Diff.	SE of diff.	n1	n2	q	DF
V vs. L	0.2486	0.7217	-0.4731	0.06927	20	20	9.658	53

V vs. F+L	0.2486	0.2996	-0.05103	0.07348	20	16	0.9821	53
L vs. F+L	0.7217	0.2996	0.4221	0.07348	20	16	8.124	53

Figure 4d Immunofluorescence staining: Iba-1 % areafraction CA1

Number of families	1							
Number of comparisons per family	3							
Alpha	0.05							
Tukey's multiple comparisons test	Mean Diff.	95.00% CI of diff.	Significant ?	Summary	Adjusted P Value			
V vs. L	-0.2605	-0.4489 to -0.07196	Yes	**	0.0044	E-F		
V vs. F+L	-0.08231	-0.2789 to 0.1143	No	ns	0.5746	E-G		
L vs. F+L	0.1781	-0.01848 to 0.3748	No	ns	0.0832	F-G		
Test details	Mean 1	Mean 2	Mean Diff.	SE of diff.	n1	n2	q	DF
V vs. L	0.2934	0.5539	-0.2605	0.07821	20	20	4.709	54
V vs. F+L	0.2934	0.3757	-0.08231	0.08159	20	17	1.427	54
L vs. F+L	0.5539	0.3757	0.1781	0.08159	20	17	3.088	54

Figure 4d Immunofluorescence staining: Iba-1 % area fraction -DG

Number of families	1							
Number of comparisons per family	3							
Alpha	0.05							
Tukey's multiple comparisons test	Mean Diff.	95.00% CI of diff.	Significant ?	Summary	Adjusted P Value			
V vs. L	-0.3313	-0.5168 to -0.1458	Yes	***	0.0002	I-J		
V vs. F+L	-0.1632	-0.3591 to 0.03264	No	ns	0.1200	I-K		
L vs. F+L	0.1681	-0.0256 to 0.3618	No	ns	0.1012	J-K		
Test details	Mean 1	Mean 2	Mean Diff.	SE of diff.	n1	n2	q	DF
V vs. L	0.3396	0.6709	-0.3313	0.07701	20	21	6.084	55
V vs. F+L	0.3396	0.5028	-0.1632	0.08131	20	17	2.839	55
L vs. F+L	0.6709	0.5028	0.1681	0.08041	21	17	2.956	55

Figure 4d Immunofluorescence staining: Iba-1 % area fraction -CA3

Number of families	1							
Number of comparisons per family	3							
Alpha	0.05							
Tukey's multiple comparisons test	Mean Diff.	95.00% CI of diff.	Significant ?	Summary	Adjusted P Value			
V vs. L	-0.07755	-0.1853 to 0.0302	No	ns	0.2018	M-N		
V vs. F+L	-0.02286	-0.1353 to 0.08954	No	ns	0.8762	M-O		
L vs. F+L	0.05469	-0.05772 to 0.1671	No	ns	0.4746	N-O		
Test details	Mean 1	Mean 2	Mean Diff.	SE of diff.	n1	n2	q	DF
V vs. L	0.1609	0.2385	-0.07755	0.04471	20	20	2.453	54
V vs. F+L	0.1609	0.1838	-0.02286	0.04664	20	17	0.6933	54
L vs. F+L	0.2385	0.1838	0.05469	0.04664	20	17	1.658	54

Figure 5b Immunofluorescence staining: COX-2 fluorescence intensity-Cortex

Number of families	1							
Number of comparisons per family	3							
Alpha	0.05							
Tukey's multiple comparisons test	Mean Diff.	95.00% CI of diff.	Significant ?	Summary	Adjusted P Value			
V vs. L	-231	-346 to -115.9	Yes	****	<0.0001	A-B		
V vs. F+L	-8.537	-130.6 to 113.5	No	ns	0.9844	A-C		
L vs. F+L	222.4	100.4 to 344.5	Yes	***	0.0002	B-C		
Test details	Mean 1	Mean 2	Mean Diff.	SE of diff.	n1	n2	q	DF
V vs. L	100	331	-231	47.71	20	20	6.846	53
V vs. F+L	100	108.5	-8.537	50.61	20	16	0.2385	53
L vs. F+L	331	108.5	222.4	50.61	20	16	6.216	53

Figure 5b Immunofluorescence staining: COX-2 fluorescence intensity-CA1

Number of families	1							
Number of comparisons per family	3							
Alpha	0.05							
Tukey's multiple comparisons test	Mean Diff.	95.00% CI of diff.	Significant ?	Summary	Adjusted P Value			
V vs. L	-737.4	-1107 to -367.5	Yes	****	<0.0001	E-F		
V vs. F+L	-100.6	-492.9 to 291.8	No	ns	0.8109	E-G		
L vs. F+L	636.9	244.5 to 1029	Yes	***	0.0008	F-G		
Test details	Mean 1	Mean 2	Mean Diff.	SE of diff.	n1	n2	q	DF
V vs. L	100	837.4	-737.4	153.4	20	20	6.798	53
V vs. F+L	100	200.6	-100.6	162.7	20	16	0.874	53
L vs. F+L	837.4	200.6	636.9	162.7	20	16	5.535	53

Figure 5b Immunofluorescence staining: COX-2 fluorescence intensity-DG

Number of families	1							
Number of comparisons per family	3							
Alpha	0.05							
Tukey's multiple comparisons test	Mean Diff.	95.00% CI of diff.	Significant ?	Summary	Adjusted P Value			
V vs. L	-1265	-2144 to -386.1	Yes	**	0.0029	A-B		
V vs. F+L	3.195	-929.1 to 935.5	No	ns	>0.9999	A-C		
L vs. F+L	1268	336 to 2201	Yes	**	0.0051	B-C		
Test details	Mean 1	Mean 2	Mean Diff.	SE of diff.	n1	n2	q	DF
V vs. L	100	1365	-1265	364.5	20	20	4.908	53
V vs. F+L	100	96.81	3.195	386.7	20	16	0.01168	53
L vs. F+L	1365	96.81	1268	386.7	20	16	4.639	53

Figure 5b Immunofluorescence staining: COX-2 fluorescence intensity-CA3

Number of families	1							
Number of comparisons per family	3							
Alpha	0.05							
Tukey's multiple comparisons test	Mean Diff.	95.00% CI of diff.	Significant ?	Summary	Adjusted P Value			
V vs. L	-95.39	-167.7 to -23.11	Yes	**	0.0068	E-F		
V vs. F+L	23.52	-53.14 to 100.2	No	ns	0.7410	E-G		
L vs. F+L	118.9	42.25 to 195.6	Yes	**	0.0013	F-G		
Test details	Mean 1	Mean 2	Mean Diff.	SE of diff.	n1	n2	q	DF
V vs. L	100	195.4	-95.39	29.98	20	20	4.5	53
V vs. F+L	100	76.48	23.52	31.79	20	16	1.046	53
L vs. F+L	195.4	76.48	118.9	31.79	20	16	5.289	53

Figure 5d Immunofluorescence staining: IL-1β fluorescence intensity-Cortex

Number of families	1							
Number of comparisons per family	3							
Alpha	0.05							
Tukey's multiple comparisons test	Mean Diff.	95.00% CI of diff.	Significant ?	Summary	Adjusted P Value			
V vs. L	-685.4	-1032 to -339	Yes	****	<0.0001	A-B		
V vs. F+L	9.574	-336.8 to 356	No	ns	0.9975	A-C		
L vs. F+L	695	360.3 to 1030	Yes	****	<0.0001	B-C		
Test details	Mean 1	Mean 2	Mean Diff.	SE of diff.	n1	n2	q	DF
V vs. L	100	785.4	-685.4	142.7	14	16	6.792	43
V vs. F+L	100	90.43	9.574	142.7	14	16	0.09488	43
L vs. F+L	785.4	90.43	695	137.9	16	16	7.129	43

Figure 5d Immunofluorescence staining: IL-1β fluorescence intensity-CA1

Number of families	1							
Number of comparisons per family	3							
Alpha	0.05							
Tukey's multiple comparisons test	Mean Diff.	95.00% CI of diff.	Significant ?	Summary	Adjusted P Value			
V vs. L	-274.9	-445.8 to -104.1	Yes	***	0.0009	E-F		
V vs. F+L	41.97	-128.9 to 212.8	No	ns	0.8229	E-G		

L vs. F+L	316.9	151.8 to 482	Yes	****	<0.0001	F-G		
Test details	Mean 1	Mean 2	Mean Diff.	SE of diff.	n1	n2	q	DF
V vs. L	100	374.9	-274.9	70.39	14	16	5.523	43
V vs. F+L	100	58.03	41.97	70.39	14	16	0.8433	43
L vs. F+L	374.9	58.03	316.9	68.01	16	16	6.59	43

Figure 5d Immunofluorescence staining: IL-1β fluorescence intensity-DG

Number of families	1							
Number of comparisons per family	3							
Alpha	0.05							
Tukey's multiple comparisons test	Mean Diff.	95.00% CI of diff.	Significant ?	Summary	Adjusted P Value			
V vs. L	-222.7	-407.3 to -38.13	Yes	*	0.0147	A-B		
V vs. F+L	58.25	-126.3 to 242.8	No	ns	0.7256	A-C		
L vs. F+L	281	102.6 to 459.3	Yes	**	0.0012	B-C		
Test details	Mean 1	Mean 2	Mean Diff.	SE of diff.	n1	n2	q	DF
V vs. L	100	322.7	-222.7	76.04	14	16	4.142	43
V vs. F+L	100	41.75	58.25	76.04	14	16	1.083	43
L vs. F+L	322.7	41.75	281	73.46	16	16	5.409	43

Figure 5d Immunofluorescence staining: IL-1β fluorescence intensity-CA3

Number of families	1							
Number of comparisons per family	3							
Alpha	0.05							
Tukey's multiple comparisons test	Mean Diff.	95.00% CI of diff.	Significant ?	Summary	Adjusted P Value			
V vs. L	-284.3	-478.3 to -90.32	Yes	**	0.0026	E-F		
V vs. F+L	59.59	-134.4 to 253.6	No	ns	0.7379	E-G		
L vs. F+L	343.9	156.5 to 531.3	Yes	***	0.0002	F-G		
Test details	Mean 1	Mean 2	Mean Diff.	SE of diff.	n1	n2	q	DF
V vs. L	100	384.3	-284.3	79.92	14	16	5.031	43
V vs. F+L	100	40.41	59.59	79.92	14	16	1.054	43
L vs. F+L	384.3	40.41	343.9	77.21	16	16	6.299	43

Figure 6b Immunofluorescence staining: Iba-1 fluorescence intensity-Cortex

Number of families	1							
Number of comparisons per family	3							
Alpha	0.05							
Tukey's multiple comparisons test	Mean Diff.	95.00% CI of diff.	Significant?	Summary	Adjusted P Value			
V vs. L	-25.01	-45.59 to -4.436	Yes	*	0.0132	A-B		
V vs. F+L	-0.6908	-21.71 to 20.33	No	ns	0.9966	A-C		
L vs. F+L	24.32	2.936 to 45.71	Yes	*	0.0219	B-C		
Test details	Mean 1	Mean 2	Mean Diff.	SE of diff.	n1	n2	q	DF
V vs. L	100	125	-25.01	8.606	28	26	4.111	75
V vs. F+L	100	100.7	-0.6908	8.79	28	24	0.111<br>1	75
L vs. F+L	125	100.7	24.32	8.945	26	24	3.846	75

Figure 6b Immunofluorescence staining: Iba-1 fluorescence intensity-CA1

Number of families	1							
Number of comparisons per family	3							
Alpha	0.05							
Tukey's multiple comparisons test	Mean Diff.	95.00% CI of diff.	Significant ?	Summary	Adjusted P Value			
V vs. L	-18.32	-33.98 to -2.669	Yes	*	0.0177	E-F		
V vs. F+L	-2.532	-17.87 to 12.81	No	ns	0.9177	E-G		
L vs. F+L	15.79	0.1366 to 31.44	Yes	*	0.0476	F-G		
Test details	Mean 1	Mean 2	Mean Diff.	SE of diff.	n1	n2	q	DF
V vs. L	100	118.3	-18.32	6.543	26	24	3.96	73
V vs. F+L	100	102.5	-2.532	6.411	26	26	0.5586	73
L vs. F+L	118.3	102.5	15.79	6.543	24	26	3.413	73

Figure 6b Immunofluorescence staining: Iba-1 fluorescence intensity-DG

Number of families	1							

Number of comparisons per family	3
Alpha	0.05

Tukey's multiple comparisons test	Mean Diff.	95.00% CI of diff.	Significant?	Summary	Adjusted P Value	
V vs. L	-16.49	-31.59 to -1.385	Yes	*	0.0291	I-J
V vs. F+L	0.6163	-14.18 to 15.41	No	ns	0.9945	I-K
L vs. F+L	17.1	2.001 to 32.2	Yes	*	0.0226	J-K

Test details	Mean 1	Mean 2	Mean Diff.	SE of diff.	n1	n2	q	DF
V vs. L	100	116.5	-16.49	6.312	26	24	3.694	73
V vs. F+L	100	99.38	0.6163	6.184	26	26	0.1409	73
L vs. F+L	116.5	99.38	17.1	6.312	24	26	3.832	73

Figure 6b Immunofluorescence staining: Iba-1 fluorescence intensity-CA3

Number of families	1
Number of comparisons per family	3
Alpha	0.05

Tukey's multiple comparisons test	Mean Diff.	95.00% CI of diff.	Significant?	Summary	Adjusted P Value	
V vs. L	-26.19	-48.37 to -4.004	Yes	*	0.0166	M-N
V vs. F+L	-14.19	-35.92 to 7.55	No	ns	0.2688	M-O
L vs. F+L	12	-10.18 to 34.19	No	ns	0.4029	N-O

Test details	Mean 1	Mean 2	Mean Diff.	SE of diff.	n1	n2	q	DF
V vs. L	100	126.2	-26.19	9.273	26	24	3.994	73
V vs. F+L	100	114.2	-14.19	9.086	26	26	2.208	73
L vs. F+L	126.2	114.2	12	9.273	24	26	1.83	73

Figure 6b Immunofluorescence staining: Iba-1 (+) cells/mm^2-Cortex

Number of families	1
Number of comparisons per family	3
Alpha	0.05

Tukey's multiple comparisons test	Mean Diff.	95.00% CI of diff.	Significant?	Summary	Adjusted P Value	
V vs. L	-15626943	-20298804 to -10955082	Yes	****	<0.0001	A-B
V vs. F+L	-462590	-5234319 to 4309139	No	ns	0.9708	A-C
L vs. F+L	15164354	10308658 to 20020049	Yes	****	<0.0001	B-C

Test details	Mean 1	Mean 2	Mean Diff.	SE of diff.	n1	n2	q	DF
V vs. L	9312429	24939372	-15626943	1953843	28	26	11.31	75
V vs. F+L	9312429	9775019	-462590	1995609	28	24	0.3278	75
L vs. F+L	24939372	9775019	15164354	2030725	26	24	10.56	75

Figure 6b Immunofluorescence staining: Iba-1 (+) cells/mm^2-CA1

Number of families	1
Number of comparisons per family	3
Alpha	0.05

Tukey's multiple comparisons test	Mean Diff.	95.00% CI of diff.	Significant?	Summary	Adjusted P Value	
V vs. L	-6978972	-11231796 to -2726148	Yes	***	0.0006	E-F
V vs. F+L	3158514	-1008385 to 7325414	No	ns	0.1723	E-G
L vs. F+L	10137487	5884663 to 14390311	Yes	****	<0.0001	F-G

Test details	Mean 1	Mean 2	Mean Diff.	SE of diff.	n1	n2	q	DF
V vs. L	7491560	14470532	-6978972	1777613	26	24	5.552	73
V vs. F+L	7491560	4333046	3158514	1741698	26	26	2.565	73
L vs. F+L	14470532	4333046	10137487	1777613	24	26	8.065	73

Figure 6b Immunofluorescence staining: Iba-1 (+) cells/mm^2-DG

Number of families	1
Number of comparisons per family	3
Alpha	0.05

Tukey's multiple comparisons test	Mean Diff.	95.00% CI of diff.	Significant?	Summary	Adjusted P Value			
V vs. L	-9699430	-14775875 to -4622986	Yes	****	<0.0001	I-J		
V vs. F+L	2774335	-2199544 to 7748215	No	ns	0.3809	I-K		
L vs. F+L	12473765	7397321 to 17550210	Yes	****	<0.0001	J-K		
Test details	Mean 1	Mean 2	Mean Diff.	SE of diff.	n1	n2	q	DF
V vs. L	7347759	17047189	-9699430	2121873	26	24	6.465	73
V vs. F+L	7347759	4573423	2774335	2079003	26	26	1.887	73
L vs. F+L	17047189	4573423	12473765	2121873	24	26	8.314	73

Figure 6b Immunofluorescence staining: Iba-1 (+) cells/mm^2-CA3

Number of families	1							
Number of comparisons per family	3							
Alpha	0.05							
Tukey's multiple comparisons test	Mean Diff.	95.00% CI of diff.	Significant?	Summary	Adjusted P Value			
V vs. L	-15097841	-22803887 to -7391796	Yes	****	<0.0001	M-N		
V vs. F+L	966028	-6584324 to 8516379	No	ns	0.9497	M-O		
L vs. F+L	16063869	8357824 to 23769914	Yes	****	<0.0001	N-O		
Test details	Mean 1	Mean 2	Mean Diff.	SE of diff.	n1	n2	q	DF
V vs. L	6075922	21173763	-15097841	3221005	26	24	6.629	73
V vs. F+L	6075922	5109895	966028	3155927	26	26	0.4329	73
L vs. F+L	21173763	5109895	16063869	3221005	24	26	7.053	73

Figure 6b Immunofluorescence staining: Iba-1 % area fraction -Cortex

Number of families	1							
Number of comparisons per family	3							
Alpha	0.05							
Tukey's multiple comparisons test	Mean Diff.	95.00% CI of diff.	Significant?	Summary	Adjusted P Value			
V vs. L	-1.847	-2.388 to -1.305	Yes	****	<0.0001	A-B		
V vs. F+L	-0.2392	-0.7921 to 0.3136	No	ns	0.5574	A-C		
L vs. F+L	1.607	1.045 to 2.17	Yes	****	<0.0001	B-C		
Test details	Mean 1	Mean 2	Mean Diff.	SE of diff.	n1	n2	q	DF
V vs. L	0.7797	2.626	-1.847	0.2264	28	26	11.54	75
V vs. F+L	0.7797	1.019	-0.2392	0.2312	28	24	1.463	75
L vs. F+L	2.626	1.019	1.607	0.2353	26	24	9.662	75

Figure 6b Immunofluorescence staining: Iba-1 % area fraction -CA1

Number of families	1							
Number of comparisons per family	3							
Alpha	0.05							
Tukey's multiple comparisons test	Mean Diff.	95.00% CI of diff.	Significant?	Summary	Adjusted P Value			
V vs. L	-1.133	-1.784 to -0.4817	Yes	***	0.0002	E-F		
V vs. F+L	0.1903	-0.4475 to 0.8281	No	ns	0.7561	E-G		
L vs. F+L	1.323	0.672 to 1.974	Yes	****	<0.0001	F-G		
Test details	Mean 1	Mean 2	Mean Diff.	SE of diff.	n1	n2	q	DF
V vs. L	0.7758	1.908	-1.133	0.2721	26	24	5.887	73
V vs. F+L	0.7758	0.5855	0.1903	0.2666	26	26	1.01	73
L vs. F+L	1.908	0.5855	1.323	0.2721	24	26	6.877	73

Figure 6b Immunofluorescence staining: Iba-1 % area fraction -DG

Number of families	1					
Number of comparisons per family	3					
Alpha	0.05					
Tukey's multiple comparisons test	Mean Diff.	95.00% CI of diff.	Significant?	Summary	Adjusted P Value	
V vs. L	-1.28	-1.983 to -0.5769	Yes	***	0.0001	I-J
V vs. F+L	0.08292	-0.6058 to 0.7717	No	ns	0.9553	I-K

L vs. F+L	1.363	0.6598 to 2.066	Yes	****	<0.0001	J-K		
Test details	Mean 1	Mean 2	Mean Diff.	SE of diff.	n1	n2	q	DF
V vs. L	0.7763	2.056	-1.28	0.2938	26	24	6.16	73
V vs. F+L	0.7763	0.6934	0.08292	0.2879	26	26	0.4074	73
L vs. F+L	2.056	0.6934	1.363	0.2938	24	26	6.559	73

Figure 6b Immunofluorescence staining: Iba-1 % area fraction -CA3

Number of families	1							
Number of comparisons per family	3							
Alpha	0.05							
Tukey's multiple comparisons test	Mean Diff.	95.00% CI of diff.	Significant?	Summary	Adjusted P Value			
V vs. L	-1.703	-2.587 to -0.8181	Yes	****	<0.0001	M-N		
V vs. F+L	-0.08465	-0.9513 to 0.7819	No	ns	0.9704	M-O		
L vs. F+L	1.618	0.7335 to 2.502	Yes	***	0.0001	N-O		
Test details	Mean 1	Mean 2	Mean Diff.	SE of diff.	n1	n2	q	DF
V vs. L	0.5764	2.279	-1.703	0.3697	26	24	6.513	73
V vs. F+L	0.5764	0.6611	-0.08465	0.3622	26	26	0.3305	73
L vs. F+L	2.279	0.6611	1.618	0.3697	24	26	6.189	73

Figure 6d Immunofluorescence staining: GFAP fluorescence intensity-Cortex

Number of families	1							
Number of comparisons per family	3							
Alpha	0.05							
Tukey's multiple comparisons test	Mean Diff.	95.00% CI of diff.	Significant?	Summary	Adjusted P Value			
V vs. L	-146	-223.9 to -68.07	Yes	****	<0.0001	A-B		
V vs. F+L	-24.63	-105.1 to 55.89	No	ns	0.7450	A-C		
L vs. F+L	121.3	40.83 to 201.9	Yes	**	0.0017	B-C		
Test details	Mean 1	Mean 2	Mean Diff.	SE of diff.	n1	n2	q	DF
V vs. L	100	246	-146	32.52	25	25	6.347	69
V vs. F+L	100	124.6	-24.63	33.62	25	22	1.036	69
L vs. F+L	246	124.6	121.3	33.62	25	22	5.105	69

Figure 6d Immunofluorescence staining: GFAP fluorescence intensity -CA1

Number of families	1							
Number of comparisons per family	3							
Alpha	0.05							
Tukey's multiple comparisons test	Mean Diff.	95.00% CI of diff.	Significant ?	Summary	Adjusted P Value			
V vs. L	-45.62	-88.35 to -2.876	Yes	*	0.0338	E-F		
V vs. F+L	-44.88	-87.2 to -2.559	Yes	*	0.0352	E-G		
L vs. F+L	0.7369	-41.12 to 42.59	No	ns	0.9990	F-G		
Test details	Mean 1	Mean 2	Mean Diff.	SE of diff.	n1	n2	q	DF
V vs. L	100	145.6	-45.62	17.84	23	24	3.615	69
V vs. F+L	100	144.9	-44.88	17.67	23	25	3.592	69
L vs. F+L	145.6	144.9	0.7369	17.47	24	25	0.05964	69

Figure 6d Immunofluorescence staining: GFAP fluorescence intensity -DG

Number of families	1							
Number of comparisons per family	3							
Alpha	0.05							
Tukey's multiple comparisons test	Mean Diff.	95.00% CI of diff.	Significant ?	Summary	Adjusted P Value			
V vs. L	-39.02	-89.72 to 11.68	No	ns	0.1632	I-J		
V vs. F+L	-25.37	-75.57 to 24.83	No	ns	0.4510	I-K		
L vs. F+L	13.65	-36 to 63.3	No	ns	0.7882	J-K		
Test details	Mean 1	Mean 2	Mean Diff.	SE of diff.	n1	n2	q	DF
V vs. L	100	139	-39.02	21.17	23	24	2.607	69
V vs. F+L	100	125.4	-25.37	20.96	23	25	1.712	69
L vs. F+L	139	125.4	13.65	20.73	24	25	0.9311	69

Figure 6d Immunofluorescence staining: GFAP fluorescence intensity -CA3

Number of families	1
Number of comparisons per family	3

Alpha	0.05							
Tukey's multiple comparisons test	Mean Diff.	95.00% CI of diff.	Significant?	Summary	Adjusted P Value			
V vs. L	-87.21	-167.2 to -7.229	Yes	*	0.0294	M-N		
V vs. F+L	-44.12	-123.3 to 35.07	No	ns	0.3811	M-O		
L vs. F+L	43.09	-35.24 to 121.4	No	ns	0.3903	N-O		
Test details	Mean 1	Mean 2	Mean Diff.	SE of diff.	n1	n2	q	DF
V vs. L	100	187.2	-87.21	33.39	23	24	3.694	69
V vs. F+L	100	144.1	-44.12	33.06	23	25	1.887	69
L vs. F+L	187.2	144.1	43.09	32.7	24	25	1.863	69

Figure 6d Immunofluorescence staining: GFAP (+) cells/mm^2-Cortex

Number of families	1								
Number of comparisons per family	3								
Alpha	0.05								
Tukey's multiple comparisons test	Mean Diff.	95.00% CI of diff.	Significant?	Summary	Adjusted P Value				
V vs. L	-108965	-199782 to -18148	Yes	*	0.0147	A-B			
V vs. F+L	-125402	-218133 to -32672	Yes	**	0.0052	A-C			
L vs. F+L	-16438	-108257 to 75381	No	ns	0.9038	B-C			
Test details	Mean 1	Mean 2	Mean Diff.	SE of diff.		n1	n2	q	DF
V vs. L	161961	270926	-108965	37914		24	25	4.064	69
V vs. F+L	161961	287364	-125402	38713		24	23	4.581	69
L vs. F+L	270926	287364	-16438	38333		25	23	0.6064	69

Figure 6d Immunofluorescence staining: GFAP (+) cells/mm^2-CA1

Number of families	1							
Number of comparisons per family	3							
Alpha	0.05							
Tukey's multiple comparisons test	Mean Diff.	95.00% CI of diff.	Significant?	Summary	Adjusted P Value			
V vs. L	-3649040	-5267586 to -2030495	Yes	****	<0.0001	E-F		
V vs. F+L	-3540981	-5126826 to -1955137	Yes	****	<0.0001	E-G		
L vs. F+L	108059	-1510487 to 1726605	No	ns	0.9860	F-G		
Test details	Mean 1	Mean 2	Mean Diff.	SE of diff.	n1	n2	q	DF
V vs. L	5351531	9000571	-3649040	676526	26	24	7.628	73
V vs. F+L	5351531	8892512	-3540981	662858	26	26	7.555	73
L vs. F+L	9000571	8892512	108059	676526	24	26	0.2259	73

Figure 6d Immunofluorescence staining: GFAP (+) cells/mm^2-DG

Number of families	1							
Number of comparisons per family	3							
Alpha	0.05							
Tukey's multiple comparisons test	Mean Diff.	95.00% CI of diff.	Significant?	Summary	Adjusted P Value			
V vs. L	-5946250	-9015512 to -2876988	Yes	****	<0.0001	I-J		
V vs. F+L	-2541834	-5549085 to 465416	No	ns	0.1141	I-K		
L vs. F+L	3404416	335153 to 6473678	Yes	*	0.0261	J-K		
Test details	Mean 1	Mean 2	Mean Diff.	SE of diff.	n1	n2	q	DF
V vs. L	6880460	12826709	-5946250	1282903	26	24	6.555	73
V vs. F+L	6880460	9422294	-2541834	1256983	26	26	2.86	73
L vs. F+L	12826709	9422294	3404416	1282903	24	26	3.753	73

Figure 6d Immunofluorescence staining: GFAP (+) cells/mm^2-CA3

Number of families	1					
Number of comparisons per family	3					
Alpha	0.05					
Tukey's multiple comparisons test	Mean Diff.	95.00% CI of diff.	Significant?	Summary	Adjusted P Value	
V vs. L	-7844126	-11746742 to -3941511	Yes	****	<0.0001	M-N

V vs. F+L	-3010941	-6834708 to 812825	No	ns	0.1506	M-O		
L vs. F+L	4833185	930570 to 8735800	Yes	*	0.0113	N-O		
Test details	Mean 1	Mean 2	Mean Diff.	SE of diff.	n1	n2	q	DF
V vs. L	5596338	13440464	-7844126	1631231	26	24	6.801	73
V vs. F+L	5596338	8607279	-3010941	1598274	26	26	2.664	73
L vs. F+L	13440464	8607279	4833185	1631231	24	26	4.19	73

Figure 6d Immunofluorescence staining: GFAP % area fraction -Cortex

Number of families	1							
Number of comparisons per family	3							
Alpha	0.05							
Tukey's multiple comparisons test	Mean Diff.	95.00% CI of diff.	Significant?	Summary	Adjusted P Value			
V vs. L	-7844126	-11746742 to -3941511	Yes	****	<0.0001	M-N		
V vs. F+L	-3010941	-6834708 to 812825	No	ns	0.1506	M-O		
L vs. F+L	4833185	930570 to 8735800	Yes	*	0.0113	N-O		
Test details	Mean 1	Mean 2	Mean Diff.	SE of diff.	n1	n2	q	DF
V vs. L	5596338	13440464	-7844126	1631231	26	24	6.801	73
V vs. F+L	5596338	8607279	-3010941	1598274	26	26	2.664	73
L vs. F+L	13440464	8607279	4833185	1631231	24	26	4.19	73

Figure 6d Immunofluorescence staining: GFAP % area fraction -CA1

Number of families	1							
Number of comparisons per family	3							
Alpha	0.05							
Tukey's multiple comparisons test	Mean Diff.	95.00% CI of diff.	Significant?	Summary	Adjusted P Value			
V vs. L	-7.571	-10.77 to -4.375	Yes	****	<0.0001	E-F		
V vs. F+L	-1.04	-4.171 to 2.092	No	ns	0.7076	E-G		
L vs. F+L	6.531	3.335 to 9.727	Yes	****	<0.0001	F-G		
Test details	Mean 1	Mean 2	Mean Diff.	SE of diff.	n1	n2	q	DF
V vs. L	5.242	12.81	-7.571	1.336	26	24	8.015	73
V vs. F+L	5.242	6.282	-1.04	1.309	26	26	1.124	73
L vs. F+L	12.81	6.282	6.531	1.336	24	26	6.914	73

Figure 6d Immunofluorescence staining: GFAP % area fraction -DG

Number of families	1							
Number of comparisons per family	3							
Alpha	0.05							
Tukey's multiple comparisons test	Mean Diff.	95.00% CI of diff.	Significant?	Summary	Adjusted P Value			
V vs. L	-8.509	-12.79 to -4.226	Yes	****	<0.0001	I-J		
V vs. F+L	-1.435	-5.632 to 2.761	No	ns	0.6929	I-K		
L vs. F+L	7.073	2.791 to 11.36	Yes	***	0.0005	J-K		
Test details	Mean 1	Mean 2	Mean Diff.	SE of diff.	n1	n2	q	DF
V vs. L	4.716	13.22	-8.509	1.79	26	24	6.722	73
V vs. F+L	4.716	6.152	-1.435	1.754	26	26	1.157	73
L vs. F+L	13.22	6.152	7.073	1.79	24	26	5.588	73

Figure 6d Immunofluorescence staining: GFAP % area fraction -CA3

Number of families	1							
Number of comparisons per family	3							
Alpha	0.05							
Tukey's multiple comparisons test	Mean Diff.	95.00% CI of diff.	Significant?	Summary	Adjusted P Value			
V vs. L	-7.419	-11.06 to -3.777	Yes	****	<0.0001	M-N		
V vs. F+L	-0.9875	-4.556 to 2.581	No	ns	0.7861	M-O		
L vs. F+L	6.432	2.79 to 10.07	Yes	***	0.0002	N-O		
Test details	Mean 1	Mean 2	Mean Diff.	SE of diff.	n1	n2	q	DF
V vs. L	2.506	9.925	-7.419	1.522	26	24	6.893	73
V vs. F+L	2.506	3.493	-0.9875	1.492	26	26	0.9364	73
L vs. F+L	9.925	3.493	6.432	1.522	24	26	5.975	73

Figure 7b Immunofluorescence staining: COX-2 fluorescence intensity-Cortex

Number of families	1

Number of comparisons per family	3								
Alpha	0.05								
Tukey's multiple comparisons test	Mean Diff.	95.00% CI of diff.	Significant?	Summary	Adjusted P Value				
V vs. L	-21.86	-30.52 to -13.2	Yes	****	<0.0001	A-B			
V vs. F+L	11.23	2.069 to 20.4	Yes	*	0.0125	A-C			
L vs. F+L	33.09	23.59 to 42.6	Yes	****	<0.0001	B-C			
Test details	Mean 1	Mean 2	Mean Diff.	SE of diff.	n1	n2	q	DF	
V vs. L	100	121.9	-21.86	3.608	26	22	8.569	63	
V vs. F+L	100	88.77	11.23	3.819	26	18	4.161	63	
L vs. F+L	121.9	88.77	33.09	3.958	22	18	11.82	63	

Figure 7b Immunofluorescence staining: COX-2 fluorescence intensity-CA1

Number of families	1								
Number of comparisons per family	3								
Alpha	0.05								
Tukey's multiple comparisons test	Mean Diff.	95.00% CI of diff.	Significant ?	Summary	Adjusted P Value				
V vs. L	-10.44	-19.34 to -1.537	Yes	*	0.0176	E-F			
V vs. F+L	8.273	-1.366 to 17.91	No	ns	0.1066	E-G			
L vs. F+L	18.71	8.907 to 28.51	Yes	****	<0.0001	F-G			
Test details	Mean 1	Mean 2	Mean Diff.	SE of diff.	n1	n2	q	DF	
V vs. L	100	110.4	-10.44	3.71	26	24	3.978	65	
V vs. F+L	100	91.73	8.273	4.019	26	18	2.911	65	
L vs. F+L	110.4	91.73	18.71	4.087	24	18	6.474	65	

Figure 7b Immunofluorescence staining: COX-2 fluorescence intensity-DG

Number of families	1								
Number of comparisons per family	3								
Alpha	0.05								
Tukey's multiple comparisons test	Mean Diff.	95.00% CI of diff.	Significant?	Summary	Adjusted P Value				
Veh vs. LPS	-21.86	-30.52 to -13.2	Yes	****	<0.0001	A-B			
Veh vs. LPS FEL	11.23	2.069 to 20.4	Yes	*	0.0125	A-C			
LPS vs. LPS FEL	33.09	23.59 to 42.6	Yes	****	<0.0001	B-C			
Test details	Mean 1	Mean 2	Mean Diff.	SE of diff.	n1	n2	q	DF	
Veh vs. LPS	100	121.9	-21.86	3.608	26	22	8.569	63	
Veh vs. LPS FEL	100	88.77	11.23	3.819	26	18	4.161	63	
LPS vs. LPS FEL	121.9	88.77	33.09	3.958	22	18	11.82	63	

Figure 7b Immunofluorescence staining: COX-2 fluorescence intensity-CA3

Number of families	1								
Number of comparisons per family	3								
Alpha	0.05								
Tukey's multiple comparisons test	Mean Diff.	95.00% CI of diff.	Significant ?	Summary	Adjusted P Value				
Veh vs. LPS	-10.44	-19.34 to -1.537	Yes	*	0.0176	E-F			
Veh vs. LPS FEL	8.273	-1.366 to 17.91	No	ns	0.1066	E-G			
LPS vs. LPS FEL	18.71	8.907 to 28.51	Yes	****	<0.0001	F-G			
Test details	Mean 1	Mean 2	Mean Diff.	SE of diff.	n1	n2	q	DF	
Veh vs. LPS	100	110.4	-10.44	3.71	26	24	3.978	65	
Veh vs. LPS FEL	100	91.73	8.273	4.019	26	18	2.911	65	
LPS vs. LPS FEL	110.4	91.73	18.71	4.087	24	18	6.474	65	

Figure 7d Immunofluorescence staining: IL-1β fluorescence intensity-Cortex

Number of families	1
Number of comparisons per family	3

Alpha	0.05								
Tukey's multiple comparisons test	Mean Diff.	95.00% CI of diff.	Significant?	Summary	Adjusted P Value				
V vs. L	-225.1	-286.9 to -163.3	Yes	****	<0.0001	A-B			
V vs. F+L	-78.25	-138.6 to -17.95	Yes	**	0.0076	A-C			
L vs. F+L	146.8	81.81 to 211.8	Yes	****	<0.0001	B-C			
Test details	Mean 1	Mean 2	Mean Diff.	SE of diff.	n1	n2	q	DF	
V vs. L	100	325.1	-225.1	25.83	30	22	12.32	73	
V vs. F+L	100	178.3	-78.25	25.2	30	24	4.391	73	
L vs. F+L	325.1	178.3	146.8	27.17	22	24	7.642	73	

Figure 7d Immunofluorescence staining: IL-1β fluorescence intensity-CA1

Number of families	1							
Number of comparisons per family	3							
Alpha	0.05							
Tukey's multiple comparisons test	Mean Diff.	95.00% CI of diff.	Significant?	Summary	Adjusted P Value			
Veh vs. LPS	-225.1	-286.9 to -163.3	Yes	****	<0.0001	A-B		
Veh vs. LPS FEL	-78.25	-138.6 to -17.95	Yes	**	0.0076	A-C		
LPS vs. LPS FEL	146.8	81.81 to 211.8	Yes	****	<0.0001	B-C		
Test details	Mean 1	Mean 2	Mean Diff.	SE of diff.	n1	n2	q	DF
Veh vs. LPS	100	325.1	-225.1	25.83	30	22	12.32	73
Veh vs. LPS FEL	100	178.3	-78.25	25.2	30	24	4.391	73
LPS vs. LPS FEL	325.1	178.3	146.8	27.17	22	24	7.642	73

Figure 7d Immunofluorescence staining: IL-1β fluorescence intensity-DG

Number of families	1							
Number of comparisons per family	3							
Alpha	0.05							
Tukey's multiple comparisons test	Mean Diff.	95.00% CI of diff.	Significant?	Summary	Adjusted P Value			
Veh vs. LPS	-225.1	-286.9 to -163.3	Yes	****	<0.0001	A-B		
Veh vs. LPS FEL	-78.25	-138.6 to -17.95	Yes	**	0.0076	A-C		
LPS vs. LPS FEL	146.8	81.81 to 211.8	Yes	****	<0.0001	B-C		
Test details	Mean 1	Mean 2	Mean Diff.	SE of diff.	n1	n2	q	DF
Veh vs. LPS	100	325.1	-225.1	25.83	30	22	12.32	73
Veh vs. LPS FEL	100	178.3	-78.25	25.2	30	24	4.391	73
LPS vs. LPS FEL	325.1	178.3	146.8	27.17	22	24	7.642	73

Figure 7d Immunofluorescence staining: IL-1β fluorescence intensity-CA3

Number of families	1							
Number of comparisons per family	3							
Alpha	0.05							
Tukey's multiple comparisons test	Mean Diff.	95.00% CI of diff.	Significant ?	Summary	Adjusted P Value			
Veh vs. LPS	-212.4	-275 to -149.8	Yes	****	<0.0001	E-F		
Veh vs. LPS FEL	-90.84	-154.9 to -26.74	Yes	**	0.0032	E-G		
LPS vs. LPS FEL	121.6	55.72 to 187.5	Yes	***	0.0001	F-G		
Test details	Mean 1	Mean 2	Mean Diff.	SE of diff.	n1	n2	q	DF
Veh vs. LPS	100	312.4	-212.4	26.14	27	24	11.49	70
Veh vs. LPS FEL	100	190.8	-90.84	26.77	27	22	4.799	70
LPS vs. LPS FEL	312.4	190.8	121.6	27.51	24	22	6.251	70

Figure 8a Y-maze (Spontaneous alteration): Normal distribution test

Number of values	10	11	10

Minimum	58	46	50
25% Percentile	61.75	57	65.88
Median	70	58	72.5
75% Percentile	74.5	65	80.5
Maximum	82	75	85.7
Mean	69.31	59.46	71.72
Std. Deviation	7.764	7.868	10.37
Std. Error of Mean	2.455	2.372	3.28
Lower 95% CI of mean	63.76	54.18	64.3
Upper 95% CI of mean	74.86	64.75	79.14
Sum	693.1	654.1	717.2
D'Agostino & Pearson normality test			
K2	0.1618	0.7809	2.354
P value	0.9223	0.6768	0.3083
Passed normality test (alpha=0.05)?	Yes	Yes	Yes
P value summary	ns	ns	ns

Figure 8a Y-maze (Spontaneous alteration): One-way ANOVA

Number of families	1							
Number of comparisons per family	3							
Alpha	0.05							
Tukey's multiple comparisons test	Mean Diff.	95.00% CI of diff.	Significant ?	Summary	Adjusted P Value			
V vs. L	9.846	0.4174 to 19.28	Yes	*	0.0393	A-B		
V vs. F+L	-2.41	-12.06 to 7.241	No	ns	0.8116	A-C		
L vs. F+L	-12.26	-21.69 to -2.827	Yes	**	0.0089	B-C		
Test details	Mean 1	Mean 2	Mean Diff.	SE of diff.	n1	n2	q	DF
V vs. L	69.31	59.46	9.846	3.811	10	11	3.654	28
V vs. F+L	69.31	71.72	-2.41	3.9	10	10	0.8738	28
L vs. F+L	59.46	71.72	-12.26	3.811	11	10	4.549	28

Figure 8a Y-maze (Number of total entry): One-way ANOVA

Number of families	1							
Number of comparisons per family	3							
Alpha	0.05							
Tukey's multiple comparisons test	Mean Diff.	95.00% CI of diff.	Significant ?	Summary	Adjusted P Value			
V vs. L	1.4	-3.596 to 6.396	No	ns	0.7693	A-B		
V vs. F+L	1.2	-3.914 to 6.314	No	ns	0.8315	A-C		
L vs. F+L	-0.2	-5.196 to 4.796	No	ns	0.9946	B-C		
Test details	Mean 1	Mean 2	Mean Diff.	SE of diff.	n1	n2	q	DF
V vs. L	17.4	16	1.4	2.019	10	11	0.9805	28
V vs. F+L	17.4	16.2	1.2	2.067	10	10	0.8211	28
L vs. F+L	16	16.2	-0.2	2.019	11	10	0.1401	28

Figure 8b NOR (test): Normal distribution test

Number of values	10	7	8
Minimum	33.63	25.88	39.03
25% Percentile	54.28	27.24	58.63
Median	71.35	36.64	73.61
75% Percentile	78.83	68.87	86.54
Maximum	87.96	82.94	95.38
Mean	67.16	45.8	71.69
Std. Deviation	16.86	22.09	18.05
Std. Error of Mean	5.332	7.812	6.383
Lower 95% CI of mean	55.1	27.33	56.6
Upper 95% CI of mean	79.22	64.27	86.78
Sum	671.6	366.4	573.5
D'Agostino & Pearson normality test			
K2	1.865	1.743	0.8822
P value	0.3936	0.4182	0.6433
Passed normality test (alpha=0.05)?	Yes	Yes	Yes
P value summary	ns	ns	ns

Figure 8b NOR (Training): One-way ANOVA

Number of families	1
Number of comparisons per family	3

Alpha	0.05							
Tukey's multiple comparisons test	Mean Diff.	95.00% CI of diff.	Significant ?	Summary	Adjusted P Value			
V vs. L	3.317	-7.732 to 14.37	No	ns	0.7356	A-B		
V vs. F+L	-1.102	-11.4 to 9.199	No	ns	0.9613	A-C		
L vs. F+L	-4.419	-15.72 to 6.88	No	ns	0.5969	B-C		
Test details	Mean 1	Mean 2	Mean Diff.	SE of diff.	n1	n2	q	DF
V vs. L	49.83	46.52	3.317	4.412	10	7	1.063	23
V vs. F+L	49.83	50.93	-1.102	4.113	10	9	0.3788	23
L vs. F+L	46.52	50.93	-4.419	4.512	7	9	1.385	23

Figure 8b NOR (Test): One-way ANOVA

Number of families	1							
Number of comparisons per family	3							
Alpha	0.05							
Tukey's multiple comparisons test	Mean Diff.	95.00% CI of diff.	Significant ?	Summary	Adjusted P Value			
V vs. L	18.51	-4.802 to 41.83	No	ns	0.1371	D-E		
V vs. F+L	-4.529	-26.97 to 17.91	No	ns	0.8688	D-F		
L vs. F+L	-23.04	-47.53 to 1.444	No	ns	0.0676	E-F		
Test details	Mean 1	Mean 2	Mean Diff.	SE of diff.	n1	n2	q	DF
V vs. L	67.16	48.65	18.51	9.282	10	7	2.821	22
V vs. F+L	67.16	71.69	-4.529	8.934	10	8	0.7169	22
L vs. F+L	48.65	71.69	-23.04	9.748	7	8	3.343	22

Figure 8d Golgi staining: AO

Number of families	1							
Number of comparisons per family	3							
Alpha	0.05							
Tukey's multiple comparisons test	Mean Diff.	95.00% CI of diff.	Significant?	Summary	Adjusted P Value			
V vs. L	2.4	-1.016 to 5.815	No	ns	0.2166	A-B		
V vs. F+L	2.73	-0.4382 to 5.898	No	ns	0.1041	A-C		
L vs. F+L	0.3302	-3.048 to 3.708	No	ns	0.9698	B-C		
Test details	Mean 1	Mean 2	Mean Diff.	SE of diff.	n1	n2	q	DF
V vs. L	24.36	21.96	2.4	1.415	19	15	2.398	51
V vs. F+L	24.36	21.63	2.73	1.312	19	20	2.942	51
L vs. F+L	21.96	21.63	0.3302	1.399	15	20	0.3337	51

Figure 8d Golgi staining: BS

Number of families	1							
Number of comparisons per family	3							
Alpha	0.05							
Tukey's multiple comparisons test	Mean Diff.	95.00% CI of diff.	Significant?	Summary	Adjusted P Value			
V vs. L	4.162	0.8461 to 7.478	Yes	*	0.0107	D-E		
V vs. F+L	0.8005	-2.37 to 3.971	No	ns	0.8145	D-F		
L vs. F+L	-3.361	-6.587 to -0.1359	Yes	*	0.0394	E-F		
Test details	Mean 1	Mean 2	Mean Diff.	SE of diff.	n1	n2	q	DF
V vs. L	26.3	22.14	4.162	1.369	16	15	4.299	46
V vs. F+L	26.3	25.5	0.8005	1.309	16	18	0.8649	46
L vs. F+L	22.14	25.5	-3.361	1.332	15	18	3.569	46

Figure 8f Immunofluorescence staining: Iba-1 fluorescence intensity-Cortex

Number of families	1							
Number of comparisons per family	3							
Alpha	0.05							
Tukey's multiple comparisons test	Mean Diff.	95.00% CI of diff.	Significant ?	Summary	Adjusted P Value			
V vs. L	-67.47	-132.3 to -2.598	Yes	*	0.0399	A-B		
V vs. F+L	52.83	-12.04 to 117.7	No	ns	0.1305	A-C		
L vs. F+L	120.3	55.42 to 185.2	Yes	***	0.0001	B-C		
Test details	Mean 1	Mean 2	Mean Diff.	SE of diff.	n1	n2	q	DF
V vs. L	100	167.5	-67.47	26.77	16	16	3.565	45
V vs. F+L	100	47.17	52.83	26.77	16	16	2.791	45

L vs. F+L	167.5	47.17	120.3	26.77		16	16	6.356	45
**Figure 8f Immunofluorescence staining: Iba-1 fluorescence intensity-CA1**									
Number of families	1								
Number of comparisons per family	3								
Alpha	0.05								
Tukey's multiple comparisons test	Mean Diff.	95.00% CI of diff.	Significant?	Summary	Adjusted P Value				
V vs. L	-51.59	-112.1 to 8.883	No	ns	0.1082	E-F			
V vs. F+L	20.83	-39.64 to 81.3	No	ns	0.6835	E-G			
L vs. F+L	72.42	11.95 to 132.9	Yes	*	0.0155	F-G			
Test details	Mean 1	Mean 2	Mean Diff.	SE of diff.		n1	n2	q	DF
V vs. L	100	151.6	-51.59	24.95		16	16	2.924	45
V vs. F+L	100	79.17	20.83	24.95		16	16	1.181	45
L vs. F+L	151.6	79.17	72.42	24.95		16	16	4.105	45
**Figure 8f Immunofluorescence staining: Iba-1 fluorescence intensity-DG**									
Number of families	1								
Number of comparisons per family	3								
Alpha	0.05								
Tukey's multiple comparisons test	Mean Diff.	95.00% CI of diff.	Significant?	Summary	Adjusted P Value				
V vs. L	-71.73	-147 to 3.587	No	ns	0.0649	I-J			
V vs. F+L	40.39	-34.93 to 115.7	No	ns	0.4028	I-K			
L vs. F+L	112.1	36.8 to 187.4	Yes	**	0.0022	J-K			
Test details	Mean 1	Mean 2	Mean Diff.	SE of diff.		n1	n2	q	DF
V vs. L	100	171.7	-71.73	31.08		16	16	3.264	45
V vs. F+L	100	59.61	40.39	31.08		16	16	1.838	45
L vs. F+L	171.7	59.61	112.1	31.08		16	16	5.102	45
**Figure 8f Immunofluorescence staining: Iba-1 fluorescence intensity-CA3**									
Number of families	1								
Number of comparisons per family	3								
Alpha	0.05								
Tukey's multiple comparisons test	Mean Diff.	95.00% CI of diff.	Significant?	Summary	Adjusted P Value				
V vs. L	-74.13	-132.3 to -15.99	Yes	**	0.0094	M-N			
V vs. F+L	7.36	-50.78 to 65.5	No	ns	0.9495	M-O			
L vs. F+L	81.49	23.35 to 139.6	Yes	**	0.0040	N-O			
Test details	Mean 1	Mean 2	Mean Diff.	SE of diff.		n1	n2	q	DF
V vs. L	100	174.1	-74.13	23.99		16	16	4.37	45
V vs. F+L	100	92.64	7.36	23.99		16	16	0.4339	45
L vs. F+L	174.1	92.64	81.49	23.99		16	16	4.804	45
**Figure 8f Immunofluorescence staining: Iba-1 (+) cells/mm^2-Cortex**									
Number of families	1								
Number of comparisons per family	3								
Alpha	0.05								
Tukey's multiple comparisons test	Mean Diff.	95.00% CI of diff.	Significant?	Summary	Adjusted P Value				
V vs. L	-85.29	-198.7 to 28.11	No	ns	0.1737	A-B			
V vs. F+L	74.7	-38.7 to 188.1	No	ns	0.2576	A-C			
L vs. F+L	160	46.59 to 273.4	Yes	**	0.0038	B-C			
Test details	Mean 1	Mean 2	Mean Diff.	SE of diff.		n1	n2	q	DF
V vs. L	273.1	358.4	-85.29	46.79		16	16	2.578	45
V vs. F+L	273.1	198.4	74.7	46.79		16	16	2.258	45
L vs. F+L	358.4	198.4	160	46.79		16	16	4.836	45
**Figure 8f Immunofluorescence staining: Iba-1 (+) cells/mm^2-CA1**									
Number of families	1								
Number of comparisons per family	3								
Alpha	0.05								
Tukey's multiple comparisons test	Mean Diff.	95.00% CI of diff.	Significant?	Summary	Adjusted P Value				
V vs. L	-139.9	-267.7 to -12.18	Yes	*	0.0289	E-F			

V vs. F+L	70.54	-57.23 to 198.3	No	ns	0.3819	E-G		
L vs. F+L	210.5	82.72 to 338.3	Yes	***	0.0007	F-G		
Test details	Mean 1	Mean 2	Mean Diff.	SE of diff.	n1	n2	q	DF
V vs. L	277	416.9	-139.9	52.72	16	16	3.754	45
V vs. F+L	277	206.4	70.54	52.72	16	16	1.892	45
L vs. F+L	416.9	206.4	210.5	52.72	16	16	5.646	45

Figure 8f Immunofluorescence staining: Iba-1 (+) cells/mm^2-DG

Number of families	1							
Number of comparisons per family	3							
Alpha	0.05							
Tukey's multiple comparisons test	Mean Diff.	95.00% CI of diff.	Significant?	Summary	Adjusted P Value			
V vs. L	-161	-336.4 to 14.49	No	ns	0.0780	I-J		
V vs. F+L	89.61	-85.86 to 265.1	No	ns	0.4375	I-K		
L vs. F+L	250.6	75.12 to 426.1	Yes	**	0.0033	J-K		
Test details	Mean 1	Mean 2	Mean Diff.	SE of diff.	n1	n2	q	DF
V vs. L	303.7	464.6	-161	72.4	16	16	3.144	45
V vs. F+L	303.7	214	89.61	72.4	16	16	1.75	45
L vs. F+L	464.6	214	250.6	72.4	16	16	4.895	45

Figure 8f Immunofluorescence staining: Iba-1 (+) cells/mm^2-CA3

Number of families	1							
Number of comparisons per family	3							
Alpha	0.05							
Tukey's multiple comparisons test	Mean Diff.	95.00% CI of diff.	Significant?	Summary	Adjusted P Value			
V vs. L	-171.8	-300.9 to -42.8	Yes	**	0.0065	M-N		
V vs. F+L	-26.07	-155.1 to 103	No	ns	0.8766	M-O		
L vs. F+L	145.8	16.73 to 274.8	Yes	*	0.0235	N-O		
Test details	Mean 1	Mean 2	Mean Diff.	SE of diff.	n1	n2	q	DF
V vs. L	201.6	373.5	-171.8	53.24	16	16	4.564	45
V vs. F+L	201.6	227.7	-26.07	53.24	16	16	0.6924	45
L vs. F+L	373.5	227.7	145.8	53.24	16	16	3.872	45

Figure 8f Immunofluorescence staining: Iba-1 % area fraction -Cortex

Number of families	1							
Number of comparisons per family	3							
Alpha	0.05							
Tukey's multiple comparisons test	Mean Diff.	95.00% CI of diff.	Significant?	Summary	Adjusted P Value			
V vs. L	-0.6641	-1.084 to -0.2442	Yes	**	0.0011	A-B		
V vs. F+L	0.2541	-0.1658 to 0.6739	No	ns	0.3165	A-C		
L vs. F+L	0.9182	0.4983 to 1.338	Yes	****	<0.0001	B-C		
Test details	Mean 1	Mean 2	Mean Diff.	SE of diff.	n1	n2	q	DF
V vs. L	0.5476	1.212	-0.6641	0.1732	16	16	5.421	45
V vs. F+L	0.5476	0.2935	0.2541	0.1732	16	16	2.074	45
L vs. F+L	1.212	0.2935	0.9182	0.1732	16	16	7.495	45

Figure 8f Immunofluorescence staining: Iba-1 % area fraction -CA1

Number of families	1							
Number of comparisons per family	3							
Alpha	0.05							
Tukey's multiple comparisons test	Mean Diff.	95.00% CI of diff.	Significant?	Summary	Adjusted P Value			
V vs. L	-0.5761	-1.048 to -0.1041	Yes	*	0.0134	E-F		
V vs. F+L	0.3109	-0.1612 to 0.7829	No	ns	0.2578	E-G		
L vs. F+L	0.887	0.4149 to 1.359	Yes	***	0.0001	F-G		
Test details	Mean 1	Mean 2	Mean Diff.	SE of diff.	n1	n2	q	DF
V vs. L	0.7408	1.317	-0.5761	0.1948	16	16	4.183	45
V vs. F+L	0.7408	0.4299	0.3109	0.1948	16	16	2.257	45
L vs. F+L	1.317	0.4299	0.887	0.1948	16	16	6.44	45

Figure 8f Immunofluorescence staining: Iba-1 % area fraction -DG

Number of families	1								
Number of comparisons per family	3								
Alpha	0.05								
Tukey's multiple comparisons test	Mean Diff.	95.00% CI of diff.	Significant ?	Summary	Adjusted P Value				
V vs. L	-0.6961	-1.217 to -0.1753	Yes	**	0.0063	I-J			
V vs. F+L	0.2374	-0.2833 to 0.7582	No	ns	0.5160	I-K			
L vs. F+L	0.9335	0.4128 to 1.454	Yes	***	0.0002	J-K			
Test details	Mean 1	Mean 2	Mean Diff.	SE of diff.	n1	n2	q	DF	
V vs. L	0.6574	1.353	-0.6961	0.2149	16	16	4.581	45	
V vs. F+L	0.6574	0.4199	0.2374	0.2149	16	16	1.563	45	
L vs. F+L	1.353	0.4199	0.9335	0.2149	16	16	6.144	45	

Figure 8f Immunofluorescence staining: Iba-1 % area fraction -CA3

Number of families	1							
Number of comparisons per family	3							
Alpha	0.05							
Tukey's multiple comparisons test	Mean Diff.	95.00% CI of diff.	Significant ?	Summary	Adjusted P Value			
V vs. L	-0.4904	-0.7779 to -0.2028	Yes	***	0.0004	M-N		
V vs. F+L	0.002563	-0.285 to 0.2901	No	ns	0.9997	M-O		
L vs. F+L	0.4929	0.2054 to 0.7805	Yes	***	0.0004	N-O		
Test details	Mean 1	Mean 2	Mean Diff.	SE of diff.	n1	n2	q	DF
V vs. L	0.4523	0.9427	-0.4904	0.1187	16	16	5.845	45
V vs. F+L	0.4523	0.4498	0.002563	0.1187	16	16	0.03054	45
L vs. F+L	0.9427	0.4498	0.4929	0.1187	16	16	5.875	45
